# Rib detection using pitch-catch ultrasound and classification algorithms for a novel ultrasound therapy device

**DOI:** 10.1186/s42234-023-00127-0

**Published:** 2023-11-15

**Authors:** Claire R. W. Kaiser, Adam B. Tuma, Maryam Zebarjadi, Daniel P. Zachs, Anna J. Organ, Hubert H. Lim, Morgan N. Collins

**Affiliations:** 1 Department of Biomedical Engineering, University of Minnesota, 312 Church St. SE 7-105 Nils Hasselmo Hall, Minneapolis, MN 55455 USA; 2 Department of Otolaryngology-Head and Neck Surgery, University of Minnesota, Phillips Wangensteen Building, 516 Delaware St SE, Suite 8-240, Minneapolis, MN 55455 USA

**Keywords:** Classification, Logistic regression, Noninvasive, Portable, Pitch-catch, Rheumatoid arthritis, Ribs, Spleen, Support Vector Machine, Ultrasound

## Abstract

**Background:**

Noninvasive ultrasound (US) has been used therapeutically for decades, with applications in tissue ablation, lithotripsy, and physical therapy. There is increasing evidence that low intensity US stimulation of organs can alter physiological and clinical outcomes for treatment of health disorders including rheumatoid arthritis and diabetes. One major translational challenge is designing portable and reliable US devices that can be used by patients in their homes, with automated features to detect rib location and aid in efficient transmission of energy to organs of interest. This feasibility study aimed to assess efficacy in rib bone detection without conventional imaging, using a single channel US pitch-catch technique integrated into an US therapy device to detect pulsed US reflections from ribs.

**Methods:**

In 20 healthy volunteers, the location of the ribs and spleen were identified using a diagnostic US imaging system. Reflected ultrasound signals were recorded at five positions over the spleen and adjacent ribs using the therapy device. Signals were classified as between ribs (intercostal), partially over a rib, or fully over a rib using four models: threshold-based time domain classification, threshold-based frequency domain classification, logistic regression, and support vector machine (SVM).

**Results:**

SVM performed best overall on the All Participants cohort with accuracy up to 96.25%. All models’ accuracies were improved by separating participants into two cohorts based on Body Mass Index (BMI) and re-fitting each model. After separation into Low BMI and High BMI cohorts, a simple time-thresholding approach achieved accuracies up to 100% and 93.75%, respectively.

**Conclusion:**

These results demonstrate that US reflection signal classification can accurately provide low complexity, real-time automated onboard rib detection and user feedback to advance at-home therapeutic US delivery.

**Supplementary Information:**

The online version contains supplementary material available at 10.1186/s42234-023-00127-0.

## Background

Focused ultrasound (US) is a clinically impactful noninvasive tool for therapeutically targeting soft abdominal tissue. Thermal effects of US energy can be used to accelerate healing during physical therapy (Miller et al. 2012) or at high intensities to induce tumor necrosis for ablating cancers of the liver, kidney, pancreas, and prostate as an alternative to invasive surgery (Marberger 2005; Wu et al. 2004). Shock wave lithotripsy utilizes mechanical effects of US pressure waves to fragment urinary stones (Kudo 2022). More recently, pulsed peripheral focused ultrasound (pFUS) using lower non-destructive intensities has been shown to alter autonomic organ function, potentially via modulation of nerves projecting to the organ and/or non-neural cells within the organ (Kubanek et al. 2018; Guo et al. 2022; Yoo et al. 2022; Cotero et al. 2019). Unlike ablative US applications, these biological effects are thought to be reversible (Guo et al. 2022; Yoo et al. 2022). Our group demonstrated that daily pFUS treatments directed at the spleen significantly reduced disease severity in a mouse model of inflammatory arthritis (Zachs et al. 2019). GE Research corroborated this finding by demonstrating that pFUS directed at the hilum of the spleen triggered an anti-inflammatory response in an acute inflammatory rat sepsis model (Cotero et al. 2022). This phenomenon is thought to be caused by activation of the cholinergic anti-inflammatory pathway similar to electrical vagus nerve stimulation, and is a promising noninvasive therapy for treating inflammation, both chronic (e.g. rheumatoid arthritis) and acute (e.g. sepsis) (Borovikova et al. 2000; Tracey 2002; Huston et al. 2006; Rosas-Ballina et al. 2011; Cotero et al. 2019). Beyond inflammatory conditions, pFUS applied to the hepatoportal nerve plexus in the liver has been shown to restore glucose homeostasis in preclinical diabetic models (Cotero et al. 2019; Cotero et al. 2022) and applied to the spleen blocked ischemia/reperfusion injury (IRI) in the kidneys in preclinical IRI models (Gigliotti et al. 2013; Gigliotti et al. 2015; Inoue et al. 2019).

Translation of abdominal pFUS therapy to humans is impeded by the rib cage shielding portions of organs like the spleen, liver, and kidney. In a study targeting the liver, researchers found US intensities can be attenuated 22.7%-70.9% depending on the width and positioning of ribs (Zubair and Dickinson 2021). The distribution of rib and intercostal space widths vary widely across body types, and previously has been correlated by univariate analysis to sex, height, weight, and BMI (Kim et al. 2014). In humans, the spleen is on average 47.8% covered by left inferior ribs number 9–11, the liver 40.2% by right inferior ribs 6–11, and the kidneys 14.5% by left and right ribs 12 (Hayes et al. 2013). Therefore, consideration of rib positioning relative to these organs is necessary for proper delivery of therapeutic energy. Our group’s previous work in inflammation and splenic models drove us to focus on rib detection over the spleen for the purposes of this study, but the methods could feasibly be applied to rib detection over other organs such as the liver and kidneys.

Techniques for locating and steering around or through ribs have been developed for tissue ablation applications, but require time and resources not suitable for frequent treatments or at-home device use. These approaches typically require precise knowledge of rib location from lengthy pre-procedure imaging and depend on large transducer areas to compensate for the energy lost from deactivating (Zubair and Dickinson 2021) or diverting (Gélat et al. 2011; Aubry et al. 2008) elements to avoid rib heating. Respiration induced motion of the rib cage and organs further complicates these methods (Marquet et al. 2011). One solution is to analyze rib cage backscatter reflections, as implemented to create a rib image in real time for time reversal (Aubry et al. 2008; Marquet et al. 2011) or DORT (Cochard et al. 2011), though this approach is computationally intensive and the rib classification process was not specified.

Unlike tissue ablation, US therapy for treatment of inflammatory conditions will require frequent US sessions (e.g., daily for multiple weeks to months) (Zachs et al. 2019; Koopman et al. 2016), which will need to be performed at home by the users to be feasible. Furthermore, to enable scalability and accessibility of the treatment for a broader patient population, therapy requires a portable energy delivery device with simplified operation and appropriate user feedback for reliable patient use at home. Towards that goal, our lab is collaborating with a company partner (SecondWave Systems) that has developed a wearable device intended for delivery of US energy specifically to the spleen. The small form factor of this device could potentially support ease of manipulation by a patient or caregiver compared to large tissue ablation transducers. However, with this smaller transducer, previously described rib bypass techniques requiring complex imaging and large transducers are not feasible. Instead, precise device placement over the intercostal space is necessary for the beam to reach the spleen through the ribs. This study adds a sensing feature using a single channel received US (rUS) echo signal acquired in the center of the array to detect ribs. An accurate yet low-complexity solution to interpret and classify this rib echo signal would enable major advancements in the translation of take-home US therapy devices with lower power requirements conducive to battery-powered operation. Real-time feedback from this backscattered rUS signal can inform manual device placement by the user. If an intercostal space between ribs is detected, the US array can transmit an energy beam that is narrow enough to go through the gap or if a rib echo is detected it can direct the user to move the device to a new location.

The aim of this study was to develop rib detection and classification technology using prototype SecondWave Systems devices by applying a variety of signal processing and classification methods to single channel rUS signals acquired at different positions in relation to ribs in human participants. Commonly used machine learning algorithms for time-series classification include K-Nearest Neighbors (KNN) (Chaovalitwongse et al. 2007), Support Vector Machine (SVM) (Omondiagbe et al. 2019; Kampouraki et al. 2009), and deep learning methods such as Convolutional Neural Network (CNN) (Zhao et al. 2017) and Recurrent Neural Networks (RNN) (Ma et al. 2018). Regularization techniques (ridge, lasso), logistic regression, and thresholding techniques (Kinoshita et al. 1995; Wang et al. 2020) are also common for low complexity classification problems. The choice of classification approach depends on various factors, including the unique nature of the ultrasound signal data, dataset size, and specific classification goals. Ultimately, the methods we chose to implement were threshold-based time and frequency domain classification, and the linear classification models logistic regression and SVM. Beginning an investigation of a novel classification task with simpler methods such as thresholding and logistic regression contributes to a better understanding of the data and problem. Following this with an advanced method such as SVM, which is known for its ability to handle complex decision boundaries, can be advantageous in improving classification accuracy.

These four methods were utilized to label each signal as acquired above a rib, partially above a rib, or above an intercostal space. Efficacy of each classification method was assessed and tradeoffs in complexity of model implementation versus efficacy were considered for each task for potential use in a future take-home therapy device. Based on the results, we were able to determine that a simple time domain thresholding algorithm worked well to identify a rib when stratifying participants relative to BMI, which is encouraging for future take-home US therapy devices.

## Methods

### Ultrasound device

**Fig. 1:**
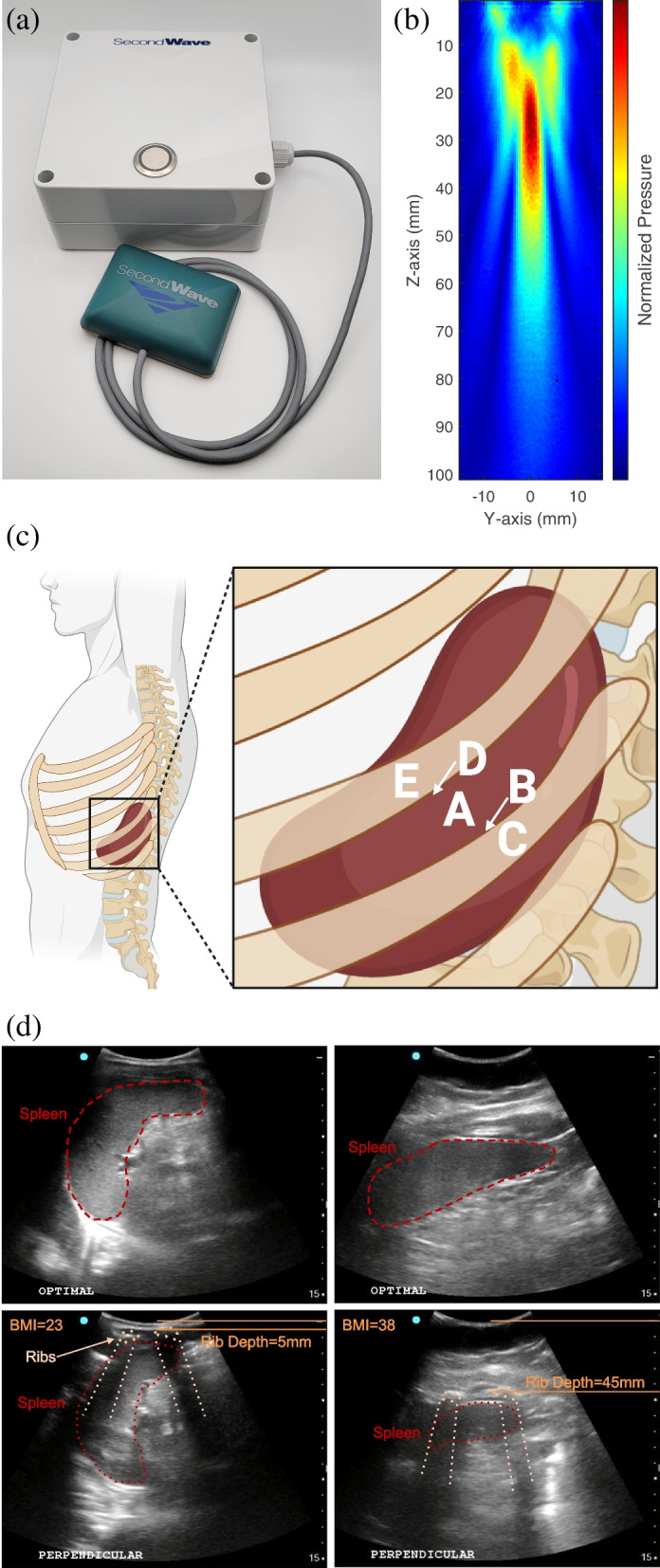
Detection of ribs over the spleen. **a** Therapeutic ultrasound device from SecondWave Systems. **b** Pressure profile of ultrasound. **c** Anatomical locations where rib echo signals were acquired: A, between ribs; B, partially over first rib; C, directly over first rib; D, partially over second rib; E, directly over second rib. Created with BioRender.com. **d** Ultrasound images collected with Sonosite Edge II: top row, transducer probe parallel to the ribs; bottom row, perpendicular to the ribs such that ribs are visible. Left column of images shows low BMI example (BMI = 23) and right column shows high BMI example (BMI = 38)

A compact, wearable, investigational device for therapeutic US energy delivery was developed by SecondWave Systems (MINI therapeutic US device, State College, PA, not commercially available), Fig. 1(a). Pulsed US is emitted by a 23.5 mm × 23.5 mm 2D grid of 256 piezoelectric elements. During transmission, elements are activated sequentially to form a cigar-shaped US beam with a high pressure point centimeters away from the surface of the transducer, Fig. 1(b). The point of highest US pressure can be dynamically focused closer to or further from the transducer by adjusting the relative timing of element activation. The beam can become highly focused or unfocused, and steered to emit at an angle from the transducer. This steering allows for specific focal point stimulation of biological targets in 3D space.

Rib detection can be implemented on this device in many configurations. For this study, our group used 248 elements to emit the transmission beam described above. A short pulse US wave (three wave cycles at 715 kHz, equating to 4.2 μs pulse width) was emitted into the body and traveled through layers of soft tissue, muscle, and ribs, if present. When the waves encountered a medium with a different acoustic impedance, a portion of the waves’ pressure was reflected back to the transducer creating an echo. The rest of the wave was absorbed at the interface, transmitted or refracted (Leighton 2007). Rib acoustic impedance is much higher than soft tissue, which resulted in a large echo signal reflected back to the transducer’s receiving element when the device was positioned over a rib. The remaining eight elements, positioned in the center of the transducer and connected together as a single channel, were used to receive rUS echo signals reflected back utilizing the pitch-catch technique. Upon receiving, the sound pressure wave was translated into an electrical voltage signal using the piezoelectric property of the receiving elements. The rUS signal and a filtered envelope signal were visualized and saved with an oscilloscope (2 Channel Digital Storage oscilloscope DSO5102P, Hantek, Shandong, China, sampling frequency 25 MHz) and analyzed later.

### Participants

This study was granted ethical approval (STUDY00013747) by the University of Minnesota Institutional Review Board on 9/13/2021. Participants provided informed written consent prior to testing. Prescreening of healthy individuals was performed over email to fill enrollment categories based on age, sex, height, and weight. Participants were ineligible if under age 18, lacking a spleen, pregnant, had breathing difficulties, were unable to maintain required seated body position, or were unable to consent due to language barriers, illiteracy, or lacking capacity. Demographic data is shown in Table 1 and Fig. S2. 20 healthy volunteers (8 Male, 12 Female) participated in the study. 19 participants identified as non-Hispanic and one as Hispanic. 19 participants identified as white and one as more than one race.

**Table 1:** Participant Demographics

	All Participants (20)	Low BMI only (12)	High BMI only (8)
Age (years)	39 ± 20 [21, 74]	36 ± 18 [22, 74]	45 ± 20 [21, 70]
Height (cm)	172 ± 10 [155, 189]	175 ± 10 [161, 189]	167 ± 9 [155, 178]
Weight (kg)	81 ± 17 [50, 109]	73 ± 13 [50, 94]	95 ± 10 [76, 109]
BMI	28 ± 6 [19, 38]	24 ± 3 [19, 28]	34 ± 2 [31, 38]
Sex	40% Male / 60% Female	50% Male / 50% Female	25% Male / 75% Female
Race	95% white / 5% more than one race	91.6% white / 8.3% more than one race	100% white
Distance to ribs (cm)	2.2 ± 1.3 [0.4, 5.3]	1.4 ± 0.7 [0.4, 3.3]	3.5 ± 0.9 [2.0, 5.3]
Distance between ribs (cm)	2.8 ± 0.9 [0.8, 4.4]	2.5 ± 0.8 [0.8, 3.6]	3.3 ± 0.7 [2.3, 4.4]

All entries are presented in the format Mean ± Standard Deviation [Minimum, Maximum]

### Testing procedure

At the beginning of the single study visit, researchers recorded demographics and physical metrics including height; weight; abdominal circumferences at the waist, navel, and the inferior tip of the sternum; length from the navel to the sternum; length from the navel to the point where the chin meets the neck; and length from the top of the hipbone to the armpit. Next, the spleen of each participant was imaged with US while sitting up at a 45° angle using the Sonosite Edge II (Fujifilm, Bothell WA, USA) to find an imaging plane parallel to the ribs where the spleen could be visualized (e.g., between ribs; Fig. 1(d) top). The imaging probe’s position was marked by a line on the skin with a non-toxic permanent marker. Then, two adjacent ribs were similarly identified via imaging and marked. US images of these three cases and an image with the probe oriented perpendicular to the ribs, providing a cross-sectional view (shown in Fig. 1(d) bottom), were saved.

Using the SecondWave Systems US device, rUS echo signals were visualized and saved on an oscilloscope in five positions relative to the ribs. First, signals were captured at the optimal position directly over the spleen and between ribs, when the rib signal was as small as possible (Fig. 1(c), location A). Moving posteriorly, signals were saved partially over the rib (Fig. 1(c), location B) and directly over the adjacent rib when the signal was highest (Fig. 1(c), location C). The process was repeated moving anteriorly (Fig. 1(c) location D and E, respectively). Ten rUS signals were saved at each of the five locations. See Supplemental Information Figure S1 for photographs of this procedure.

### Classification models

Four algorithms for signal classification of the rUS echo signals were tested with the goal of developing a technique to provide automated onboard rib classification and user feedback. rUS signals were classified into three categories: between ribs (intercostal, location A), directly over a rib (location C or E), or partially over a rib (location B or D). Four classification methods were tested: threshold-based time domain classification, threshold-based frequency domain classification, and the linear classification models logistic regression and support vector machine (SVM). The first method can be implemented in real time with analog electronics, while the latter three necessitate additional hardware and resource-intensive digital signal processing components, such as application specific integrated circuits (ASICs). Classification was carried out on data from 20 study participants each providing 10 signals acquired in 5 locations for a total of 1000 signals. 80% of the data was used for training the algorithms and the other 20% was used for testing to compute accuracy percentage and F1-score. K-fold cross validation (CV) assessment was also completed using the full dataset and average error was reported.

**Fig. 2:**
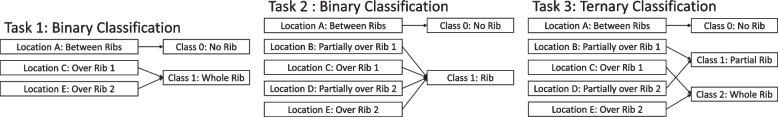
Flow chart of Tasks 1–3 including the subset of rUS signal locations used and the corresponding Class name assigned to the signal from each location

rUS signals were grouped to perform three classification tasks, as shown in Fig. 2. In Task 1, each model was trained to separate rUS signals into two distinct classes: the No Rib class (data from location A) and the Whole Rib class (data from locations C and E). In Task 2, models were again trained to separate rUS signals into two classes, the No Rib class (data from location A) and the Rib class which combined whole rib and partial rib signals (data from locations B, C, D, and E). Finally, in Task 3 models separated rUS signals into three classes: the No Rib class (data from location A), the Partial Rib class (data from locations B and D), and the Whole Rib class (data from locations C and E). In the training data for the linear classification models, the number of sample signals from each class set was balanced to avoid bias toward one class type.

Initially, each classification model was trained and tested on the full 20-participant dataset. Next, the dataset was split into two cohorts, Low BMI (12 participants) and High BMI (8 participants) based on a BMI = 30 border, which is the threshold of obesity provided by the National Institutes of Health (Pi-Sunyer 2000). Additional metrics for subdividing the participant cohorts such as ratio of abdominal circumference at the navel to height were also evaluated (see Supplementary Information), but BMI showed the highest correlation with rib depth and rib rUS time.

#### Threshold-based time domain classification

Classification using an amplitude thresholding technique was performed on time domain data. rUS signal processing included identifying the time of stimulus presentation (Time = 0) using a threshold for the initial artifact and computing the absolute value of the signal to rectify, making all voltages positive. A time window was identified that included the rib echo and excluded the initial artifact for all training data using iterative range reduction, see Table S5. Then, for Task 1 and Task 2, an amplitude threshold cutoff between the No Rib and (Whole) Rib classes was identified by minimizing error in iterative classification of the training data (Fig. S3). In Task 3, the amplitude threshold from Task 2 was used again to separate the No Rib and Rib classes. Then a second amplitude threshold was iteratively optimized to separate the Partial Rib class from the Whole Rib class. The second threshold was always a higher value than the first. The time window and amplitude threshold(s) were optimized for the All Participants dataset and then re-optimized for High BMI and Low BMI participant cohorts.

#### Threshold-based frequency domain classification

Classification using an amplitude thresholding technique was performed on frequency domain data. rUS signals were pre-processed by time windowing to eliminate the initial artifact as in time domain classification and then zero-padded with an array equal to the length of the signal. Then, a fast Fourier transform was performed and the absolute value of this frequency domain signal was computed. Finally, this frequency domain signal was windowed to eliminate frequency components below 493.8 kHz and above 931.3 kHz, see Table S5. This focused thresholding analysis on a 500 kHz band surrounding the transducer’s center frequency of 715 kHz, where the largest change in amplitude between the No Rib and Rib conditions was observed. The resulting signals were used in the three classification tasks. Frequency domain amplitude thresholds were identified by minimizing classification error iteratively using the same process described above for time domain classification. Optimization was carried out for All Participants, then the Low BMI and High BMI cohorts separately.

#### Linear classification: logistic regression and SVM

Linear models are mathematical models that use linear combinations of features to assign class labels to input data. Logistic regression assesses the class probability as a function of the linear combination of predictor features. It uses the deviance (logistic) loss function for objective-function optimization and adjustment of feature weights. In contrast, a support vector machine (SVM) assigns input data to classes using a decision boundary “hyperplane” that bisects each feature space (Omondiagbe et al. 2019). The hyperplane is chosen by maximizing the margin between the classes. Data points close to the separating hyperplane are termed support vectors.

These two linear models (logistic regression and SVM) were applied to the three classification tasks. Binary linear classification (Tasks 1 and 2) using logistic regression was carried out using the fitclinear function supplied by MATLAB (The Mathworks Inc, Natick Massachusetts USA) with a logistic regression Learner. Binary linear classification using SVM was carried out using the fitcsvm function supplied by MATLAB and a linear kernel function. For the ternary Task 3, linear classification was carried out using the fitcecoc function supplied by MATLAB with a templateLinear Learner for logistic regression and a templateSVM Learner for SVM. Input parameters for each function are reported in Table S6. Three types of features were used in the linear classifiers: time domain features, frequency domain features, and demographics/physical metric features. A maximum of 70 features were available. These models were first trained and tested on the 20 All Participants dataset, then the Low BMI and High BMI cohorts separately.

Time domain features were derived from down-sampled rUS signals after rectification and windowing to eliminate the initial artifact. Time signals were down-sampled in bins of 100 samples by averaging over 4 μs, resulting in 23 time signal features. Separately, time signals were down-sampled in bins of 200 samples by averaging over 8 μs, resulting in 11 additional time signal features. Therefore, 34 total time domain features were used in the classifiers.

To derive frequency domain features, time domain signals were windowed, zero-padded, Fourier transformed, and rectified, as above. Frequency signals were down-sampled in bins of 10 samples by averaging over 62.5 kHz, resulting in 16 frequency domain features. Separately, frequency signals were down-sampled in bins of 20 samples by averaging over 125 kHz, resulting in 8 more frequency domain features. The peak frequency of 715 kHz, which is the transducer’s center frequency, fell in bin 6 for the frequency features computed using 125 kHz widths, and fell in bins 11 and 12 for those computed using 62.5 kHz widths. The value of the frequency signal at zero was also used as a feature because it is an average of the full signal data. Therefore, 25 total frequency domain features were used in the classifiers.

The remaining features used in the classifiers were related to participant demographics and physical metrics. Demographic features were sex and age. Physical metric features were height; weight; BMI; abdominal circumference at the waist, navel, and inferior tip of sternum; length from the navel to the sternum; length from the navel to the point where the chin meets the neck; and length from the top of the hipbone to the armpit. Therefore, 11 total demographic and physical metric features were used in the classifiers.

All features were scaled before classification such that their values fell between zero and one, for simple analysis of preferential weighting from logistic regression. Time features were scaled by 10^4^, frequency features were scaled by 10^6^, and demographic/physical metric features were scaled between 40 and 200, depending on the feature.

#### Model accuracy

Classification results are presented in terms of accuracy percentage of correctly classified signals and in terms of the F1-score. Both metrics use data from the confusion matrix commonly used to define the performance of a classification algorithm. For evaluating binary accuracy (Tasks 1 and 2), the (Whole) Rib class was defined as the “positive” category and the No Rib class was the “negative” category. To calculate the accuracy, the number of true positives was divided by the total number of Whole Rib signals in the dataset and similarly the number of true negatives was divided by the total number of No Rib signals. The arithmetic mean of these two numbers was reported as the accuracy percentage of correctly classified signals. The F1-score is another way to assess the relative performance of a classifier by including the false negatives and false positives in the calculation. The F1-score is the harmonic mean of the precision ([true positives]/[true positives + false positives]) and the recall ([true positives]/[true positives + false negatives]) of the classifier. It ranges from zero to one and a higher F1-score indicates a model that has a good balance of precision and recall. The F1-score for three-classes was calculated using the macro-averaged F1-score by computing the arithmetic mean of the per-class F1-scores.

Model accuracy was also assessed using k-fold cross validation (CV) with k = 10. In this method, the data was split into 10 equal groups (folds), and 9 of 10 groups were used as training data while the final group was used as testing data. The groups were iterated through such that all 10 groups were used as testing data in one instance. The error in the classification was noted for each fold, and an average of the 10 error values was reported. This k-fold loss is the predictive inaccuracy of the classification model, with a lower loss indicating a better predictive model.

## Results

**Fig. 3:**
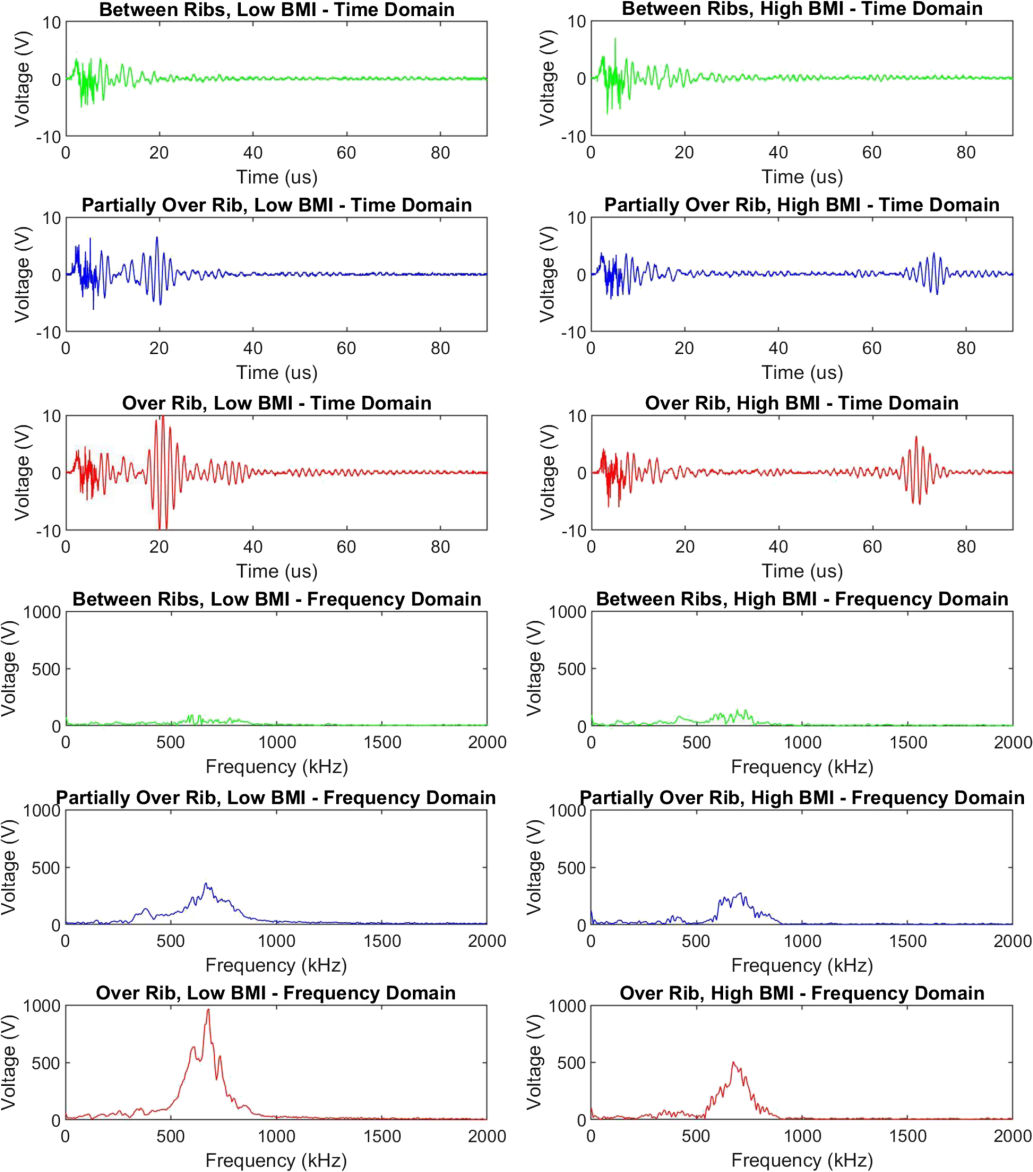
Time and frequency domain examples of acquired signals for a low BMI (22.96, left) and a high BMI (38.23, right) participant. The top six plots are rUS signals in the time domain. Signals acquired between ribs (location A, in green) have the initial artifact with no rib reflection, partially over a rib (location B and D, in blue) have small rib echoes, and directly over a rib (location C and E, in red) have larger rib reflections. The bottom six plots are rUS signals translated into the frequency domain using a fast Fourier transform (same colors apply)

Figure 3 presents typical rUS signals for three anatomical locations relative to a rib for a Low BMI (22.96, left) and High BMI (38.23, right) participant. All time domain signals begin with an initial artifact that spans about 15 μs caused by amplification of the excitation pulse and acoustic crosstalk from transmitting and receiving elements located on a single circuit board. The artifact’s shape was consistent across participants and was used as an initiation signal to indicate the Time = 0 point. A second waveform appears at 20 μs (Low BMI) or 70 μs (High BMI) indicating the US reflection from a rib. It is closer to the artifact for the Low BMI participant because the rib was closer to the skin surface thus the US took less time to reach the rib and reflect back to the transducer’s receiver. The High BMI participant’s rib signal is lower in amplitude because it traveled through more tissue resulting in wave scattering and reduced pressure measured by the receiving elements. It may have also traveled through more tissue interfaces, such as muscle and fat, causing additional reflection and absorption events that further reduced the pressure. The relationship between this rUS time signal and the time of flight distance traveled to each rib is covered in the Rib Position Analysis section. Examples of frequency domain signals are presented in the lower half of Fig. 3, and were used in threshold-based frequency domain classification, logistic regression, and SVM.

### Threshold-based time domain classification

**Fig. 4:**
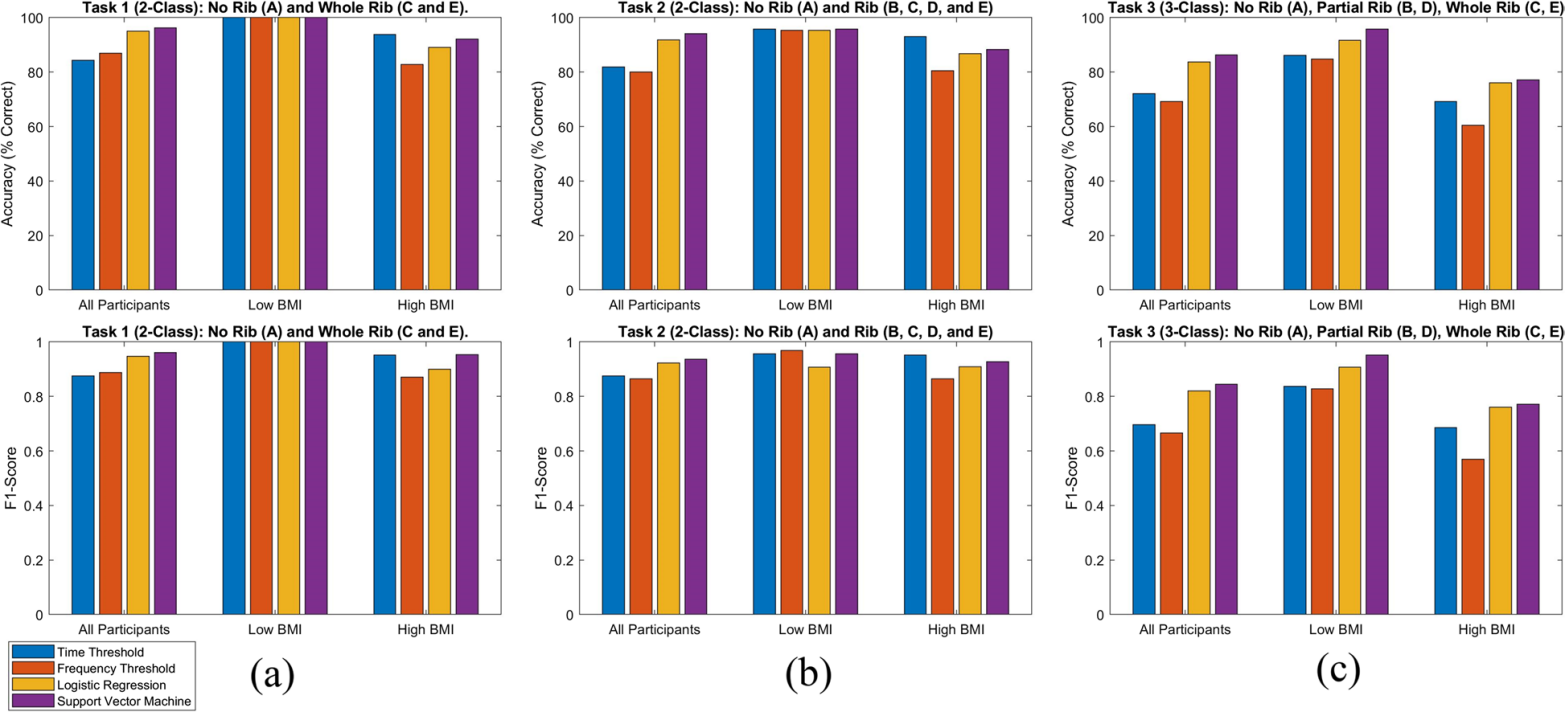
Bar graphs of classification accuracy in the top row and F1-Score in the bottom row with 20-person All participants in the left grouped bars, 12-person Low BMI cohort in centered bars, and 8-person High BMI cohort in right bars. **a** Task 1 with 2-class No Rib vs. Whole Rib. **b** Task 2 with 2-class No Rib vs. Rib. **c** Task 3 with 3-class No Rib vs. Partial Rib vs. Whole rib

The accuracy and F1-Scores of the classification models are summarized as bar plots in Fig. 4, and a complete list of these values along with CV errors are provided in Tables S1-4. Accuracy is cited below, F1-Scores and CV error followed similar trends. The blue bars in Fig. 4 indicate results of the threshold-based time domain classification. Across Tasks 1–3, when All Participants were analyzed together this model’s performance was low because the time window had to be sufficiently wide (73.84 µs) to encompass the entire range of rib echo times. Separating the two BMI cohorts allowed for smaller windows (Low BMI = 38.72 µs window; High BMI = 60.80 µs window, shifted later in time by 13.04 µs to account for longer time of flight) and more targeted amplitude thresholds (e.g., Task 1: All Participants = 2.8 V, Low BMI = 5.3 V, High BMI = 1.4 V). The BMI stratification approach yielded much more accurate classification. It minimized false negative No Rib classification, which could occur if a high amplitude threshold missed an attenuated rib echo from a deep rib. It also minimized false positive Whole Rib classification, which could occur if part of the initial artifact fell within the wide time window and was mistakenly identified as a rib. Task 3 had lower performance than the binary tasks. The majority of the classification errors originated from the separation of the Partial Rib and Whole Rib classes, which was notably less accurate for the High BMI cohort. The Low BMI cohort yielded the most accurate classification across the three tasks (1: 100%, 2: 95.83%, 3: 86.11%). Classification of the High BMI cohort (1: 93.75%, 2: 92.97%, 3: 69.17%) was more accurate than the All Participants cohort (1: 84.38%, 2: 81.88%, 3: 72.08%) for most tasks, demonstrating a significant benefit from optimizing the window and threshold parameters for BMI-specific cohorts.

### Threshold-based frequency domain classification

The accuracy and F1-Scores of threshold-based frequency domain classification are summarized in Fig. 4 as orange bars (values including CV errors are in Tables S1-4). The Low BMI cohort again yielded the most accurate classification across the three tasks (1: 100%, 2: 95.31%, 3: 84.72%). Unlike the time-thresholding case, classification of the All Participants cohort (1: 86.88%, 2: 80.00%, 3: 69.17%) was more accurate than the High BMI cohort (1: 82.81%, 2: 80.47%, 3: 60.42%) for most tasks. Most misclassifications in the All Participant group were from members of the High BMI cohort. In fact, this model applied to the High BMI cohort had the lowest performance of all classification models evaluated in this study. Though the other two groups performed on par with the time-thresholding algorithm, the lower performance on High BMI participants indicates that this specific frequency thresholding algorithm is not well suited for this application.

### Linear classification: logistic regression and SVM

Results from the logistic regression model (yellow) and the SVM (purple) using all 70 features are presented in Fig. 4. All values including CV errors are in Tables S1-4. For both models across all tasks, the Low BMI cohort was most accurate, followed by the All Participant cohort and then the High BMI cohort. For Task 1, results from the logistic regression model were 95%, 100%, and 89.06% and from the SVM were 96.25%, 100%, and 92.19% for All Participants, Low BMI, and High BMI, respectively. The analysis was repeated with fewer features, determined by the process detailed in the Supplementary Information. The classification accuracy decreased with the number of features used to train the classifier, as shown in Fig. S4(a). The Low BMI cohort was always most accurate and the High BMI cohort was least accurate. Results of Task 2 from the logistic regression model were 91.88%, 95.31%, and 86.72% and from the SVM were 94.06%, 95.83%, and 88.28% for All Participants, Low BMI, and High BMI, respectively. The accuracy was not as dependent on the number of features, Fig. S4(b). When these models were implemented with five features, each performed almost as well as with 70 features in most categories. Results of Task 3 from the logistic regression model were 83.75%, 91.67%, and 76.04% and from the SVM were 86.25%, 95.83%, and 77.08% for All Participants, Low BMI and High BMI, respectively. These models also performed well with fewer features, with peak performance around 10 features, Fig. S4(c). F1-Scores and CV error followed similar trends, and indicated that the SVM outperformed logistic regression slightly in all Tasks.

Overall, the highest classification accuracy was achieved in Task 1. Across tasks, the Low BMI cohort was more accurately classified than the High BMI cohort. The SVM model routinely performed better than the logistic regression model by a small margin (average increase in accuracy Task 1: 0.40%, Task 2: 0.59%, Task 3: 0.60%). Both models were remarkably accurate when fewer features were used. There was often a peak in accuracy at 70 features, and a second peak at 8–25 features. The SVM model was most accurate when all 70 features were used in almost all cases. The logistic regression model was often more accurate using 10–25 features than 70 features, boosting accuracy by up to 2.77%. This accuracy still did not exceed the best performance of the SVM model in these cases.

### Rib position analysis

Analysis of rUS signals containing rib echoes can provide an estimate of the rib depth by computing the time between the initial artifact and the rib signal, see Supplemental Information for details. This is the “time of flight” from the time the US wave was emitted to the time the echo reached the receiving transducer. Using the speed of sound in water (a common approximation for tissue (Gélat et al. 2011)), the time value can be converted to a distance measurement. This estimated rib depth is an accessible metric that could be internally calculated by a low-computation device. However, it is not as accurate as the rib depth measured from US images, which was considered the ground truth, Fig. S5. The median error between the two was 0.52 cm, and error was larger for participants with higher BMIs (deeper ribs), for which depth was often underestimated. In this 20-person study, the rib depth in the area over the spleen was well correlated with weight and physical metric ratios including body mass index (BMI), which is the weight in kilograms divided by height in meters squared (weight/height^2^), and the author-defined C/H ratio, which is abdominal circumference at the navel divided by height, Fig. S6. The distribution of rib width and intercostal space width as a function of age, height, weight, and BMI has previously been studied (Kim et al. 2014). For the application of delivering US therapy to the spleen, or any abdominal organ, knowledge of the intercostal space, rib depth, and organ depth are necessary for US beam targeting and estimating how much US is reaching its target.

## Discussion

This study collected rUS signals from the ribs and intercostal spaces covering the spleen of 20 healthy human participants of ranging age, sex, and body types, and classified them via four methods. In the first task that classified only No Rib or Whole Rib signals, all methods performed at or above 82% accuracy, with an F1-score ≥ 0.8710 and CV error ≤ 0.1938. The more complex linear classifiers (SVM and logistic regression) were better suited for the All Participants data set. Surprisingly, after stratification by BMI, the simplest method of time thresholding yielded very accurate results on par with the more complex models (Low BMI: 100%, F1-score 1, CV error 0.0083; High BMI: 93.75%, F1-score 0.9524, CV error 0.0050).

In the second task, signals were classified as No Rib or Rib because an algorithm that can alert the user if any type of rib echo is present (i.e., direct or partial rib blocking) will be useful for making an adjustment in device positioning. When All Participants were analyzed together, the most complex method, SVM (94%), was again most accurate. After BMI stratification, all methods except frequency thresholding performed equally well (Low BMI: > 95%, F1-score 0.9081–0.9681, CV error 0.0240–0.0521; High BMI: > 86%, F1-score ranged 0.9091–0.9516, CV error 0.0594–0.1047).

In the third task, classifiers separated signals into three classes: No Rib, Whole Rib, or Partial Rib. This could be useful information for correcting slight device placement errors in the future by automatically altering the therapeutic US beam angle to avoid a rib, rather than requiring the user to move the device. The linear classification algorithms were better suited to this task. The SVM model yielded the best accuracies across cohorts, between 77.08% (F1-score 0.7711, CV error 0.2688) and 95.83% (F1-score 0.9516, CV error 0.0500). On the other hand, the ternary task was much more difficult for the thresholding algorithms, with accuracies as low as 60.42%, F1-score ≥ 0.5694, CV error ≤ 0.4333.

There are benefits and drawbacks to each classification method depending on the task. Using All Participant’s data, simulating a situation where patient body type is not considered, the more complicated SVM linear classification model yielded the best accuracy, F1-scores, and CV errors across all tasks. Logistic regression performed second best. Interestingly, when two models were separately optimized for Low BMI and High BMI users, the time domain amplitude thresholding method performed as well or better than the linear classification methods on Tasks 1 and 2. Real-time onboard digital signal processing capabilities are necessary to implement the more complex linear classification methods, while time thresholding can be replicated using analog electronics. Therefore, the results demonstrate that when participant categorization based on easy to gather metrics is implemented, high binary classification accuracy can be achieved with simple hardware designs.

While this study captured a range of body shapes, the relatively small cohort of 20 participants is a limitation that could lead to overfitting the models to individual subjects. Additionally, most participants identified as white and non-hispanic with only one identifying as hispanic and another as more than one race. Available literature suggests race may introduce differences in skeletal (Işcan 1991; Yavuz et al. 1998; Cerezo-Román and Hernández Espinoza 2014; Meena and Rani 2014) or tissue-based features (Demerath et al. 2007). While these studies did uncover differences between races and sexes pertaining to the age at which deterioration of bones became apparent, the morphological aging changes themselves were reported to be the same for all races. Additionally, no difference in subcutaneous adipose tissue was reported between Caucasian-American/white and African-American men and the difference present in women was most pronounced around the lower abdomen, not the thoracic region where rib detection was performed. Lack of racial diversity is a limitation of this study, but given this information we would expect the age and sex diverse population that we surveyed to capture much of the variations noted due to race. Future research will include a more racially diverse population to determine the impact on rUS signal data.

In anticipation of our method’s use in this wider population, a procedure has been outlined to assess rib detection efficacy on a case-by-case basis, especially with individuals whose body features may not be fully represented in the dataset used for the model development. An envisioned initial fitting workflow for a new participant (e.g., a candidate for splenic US-based therapeutic treatment for rheumatoid arthritis) would begin with measurement of height and weight to calculate BMI followed by participant assignment to the Low BMI or High BMI cohort. Next, ultrasound imaging to locate the spleen region and ribs above would be completed using a commercial imaging system. The appropriate time domain threshold-based model would be programmed on the US device based on BMI and rib detection would be performed. If the ribs are not effectively detected, time thresholding may be reattempted with individual-specific thresholds obtained from oscilloscope measurements and efficacy of rib detection assessed. Additionally, device programming may be switched to the SVM model using in-clinic digital signal processing equipment and rib detection assessed. Data would be captured from these outlier participants and incorporated into the model for iterative refitting.

The accuracies of the linear classification methods we implemented in this study were 95.00% (logistic regression) and 96.25% (SVM) on the Task 1 All Participants binary problem, which is sufficient accuracy for our application. Future research and development, including the exploration of additional regularization techniques (ridge or lasso), advanced neural network architectures (CNN, RNN or KNN), and the collection of more extensive datasets to improve model generalization and performance can contribute to further advancements in the field of time-series analysis, particularly in the context of rib detection based on rUS signals.

Finally, understanding the relationship between rib depth, body features that can be acquired without full imaging procedures (e.g., rib depth estimate with SecondWave device, height, weight, abdominal circumference, and additional features listed in Methods), and features that require detailed imaging (e.g., ground truth rib depth, depth to target organ, intercostal space) will increase automated decision-making capabilities and reduce reliance on sophisticated and power intensive imaging. To this end, our group is developing a library of body features and imaging ground truth depths that currently includes over 100 participants across several studies. This will be a powerful screening tool to calculate the expected efficacy of spleen targeting with therapeutic US for a range of body shapes.

## Conclusion

US signals were classified using four methods across three classification tasks to indicate the presence or absence of a rib for targeted splenic US therapy. The SVM linear classification model consistently yielded the most accurate results across all tasks when analyzing all participants together. However, when participants were categorized into two BMI-based cohorts and the models were re-trained, the time domain amplitude thresholding method performed as well or better than more complex models (such as SVM) on the binary tasks. These results demonstrate the feasibility of automated onboard classification of rUS signals for rib detection using low complexity methods given basic information such as height and weight. In most clinical settings these metrics are already collected, so implementing binary classification with BMI-separated time thresholding is a practical and effective solution. For ternary classification including partial rib identification, it would be sensible to instead use SVM to ensure accurate results. These classification techniques enable reliable therapeutic US delivery to the spleen via the intercostal space using an imaging-free platform device, a method that could be developed and extended to targeting neighboring abdominal organs such as the liver or kidney. A portable US therapy device that implements rib detection as described in this paper would be uniquely suited for in-home US stimulation treatments based on portable device dimensions, reduced complexity of algorithms, and ease of use. These technologies could increase accessibility of US-based therapies for patients suffering from conditions like rheumatoid arthritis with spleen stimulation and diabetes with liver stimulation by eliminating the need for frequent in-clinic treatments by skilled professionals.

### Supplementary Information

**Additional file 1.**

## Data Availability

Supplementary information, Tables S1-S6, and Figures S1-S6 are available online. Additional data that support the findings of this study are available from the corresponding authors upon reasonable request.

## Abbreviations

BMI: Body mass index
CNN: Convolutional Neural Network
CV: Cross Validation
KNN: K-Nearest Neighbors
pFUS: Peripheral focused ultrasound
rUS: Received ultrasound
RNN: Recurrent Neural Networks
SVM: Support vector machine
US: Ultrasound
