# Supplementary material for: Rib detection using pitch-catch ultrasound and classification algorithms for a novel ultrasound therapy device
Source: Bioelectron Med. 2023 Nov 15;9:25. doi: 10.1186/s42234-023-00127-0 (PMC10647025; doi:10.1186/s42234-023-00127-0)
Supplement: Supplementary file 1 — Additional file 1. [file 42234_2023_127_MOESM1_ESM.docx]

Rib detection using pitch-catch ultrasound and classification algorithms for a novel ultrasound therapy device – Supplementary Information Document

Supplementary Tables S1-4

Tables 1-4 summarize the efficacy of the four classification models using three common metrics: accuracy percentage of correctly classified signals, F1-score, and k-fold cross-validation error. See Methods Model Accuracy section for details. In the context of these tables and the body of the paper, the “No Rib” class is used to label rUS signals that are acquired above an intercostal space (i.e. between two ribs). This class is applicable in all Tasks (1, 2, and 3). The “Whole Rib” class in Task 1 and Task 3 is used to label rUS signals that were acquired directly over a rib. The “Rib” class in Task 2 is used to label rUS signals that were acquired either directly or partially over a rib. This “Rib” class is intended to reflect the difference in the binary Task set forth in Task 2 from Task 1. The “Partial Rib” class is used to label only rUS signals acquired partially above a rib and is only used in the ternary Task 3. See Fig. 2 in the body of the paper for additional clarification.

Supplementary Tables S5-6 summarize the input parameters for the four classification models.

Supplementary Information Figures


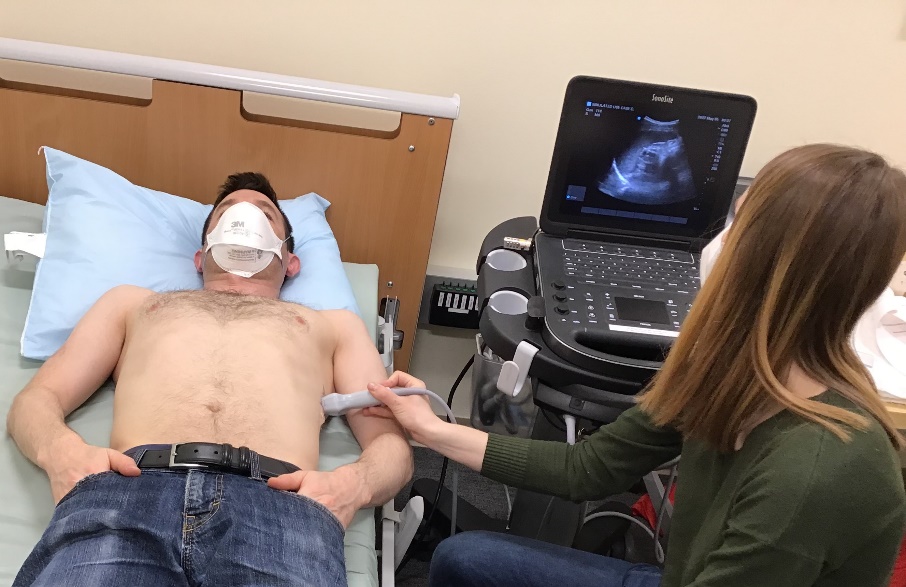

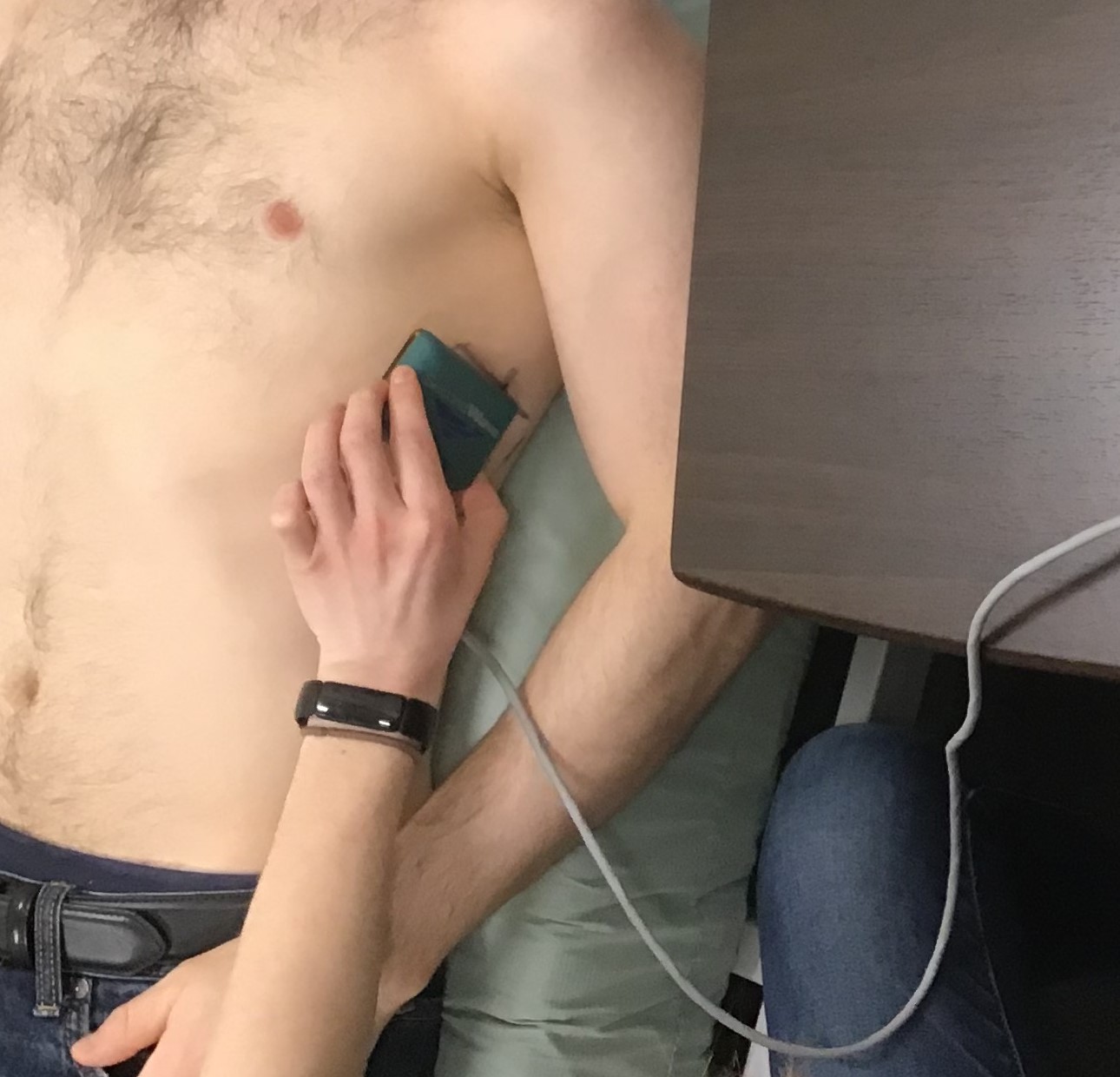

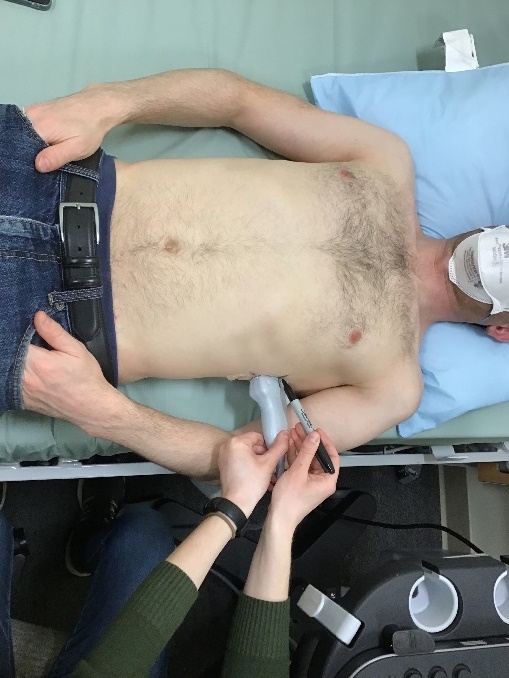

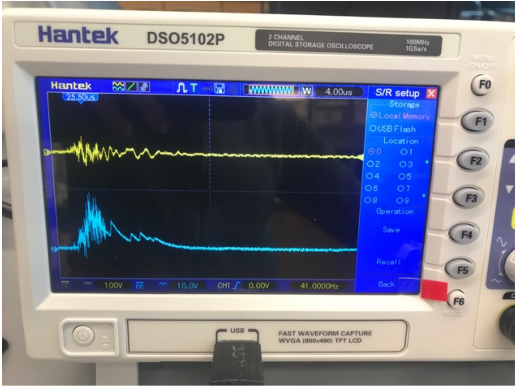

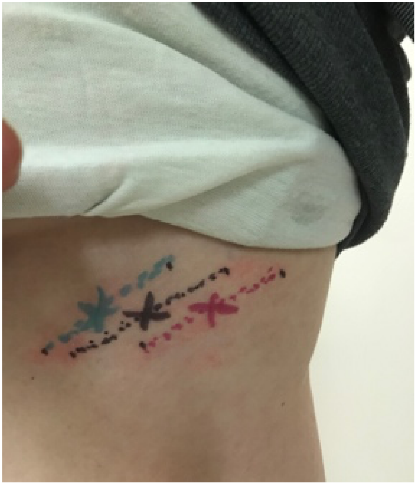


(a)

(b)

(c)

(d)

(e)

Fig. S1: Photographs of testing procedure. (a) Spleen imaging in position A (between ribs) with Sonosite Edge II diagnostic ultrasound system. (b) Marking position of the imaging transducer with a non-toxic permanent marker. (c) Final skin marking indicating position A (between ribs, black/center line), position C (over posterior rib, red/right line), and position E (over anterior rib, blue/left line). (d) Patient view of acquiring rUS echo signals in position A. (e) Oscilloscope view of acquiring unprocessed rUS signal (top/yellow) and a filtered envelope signal (bottom/blue) from position A and saving signals for analysis. Individuals in these photographs are members of the research team demonstrating study procedures and have given written consent that these photographs may be published.

| 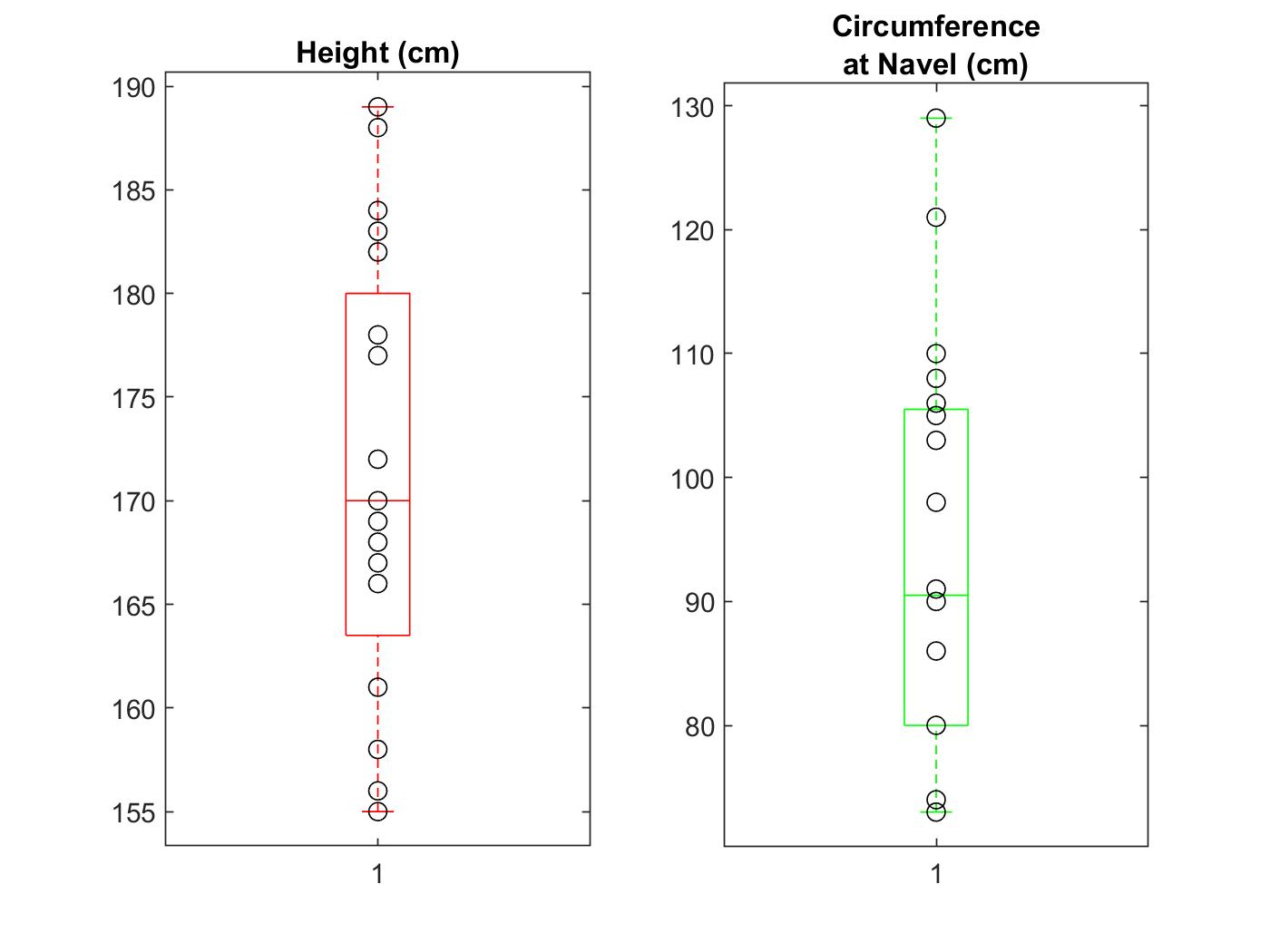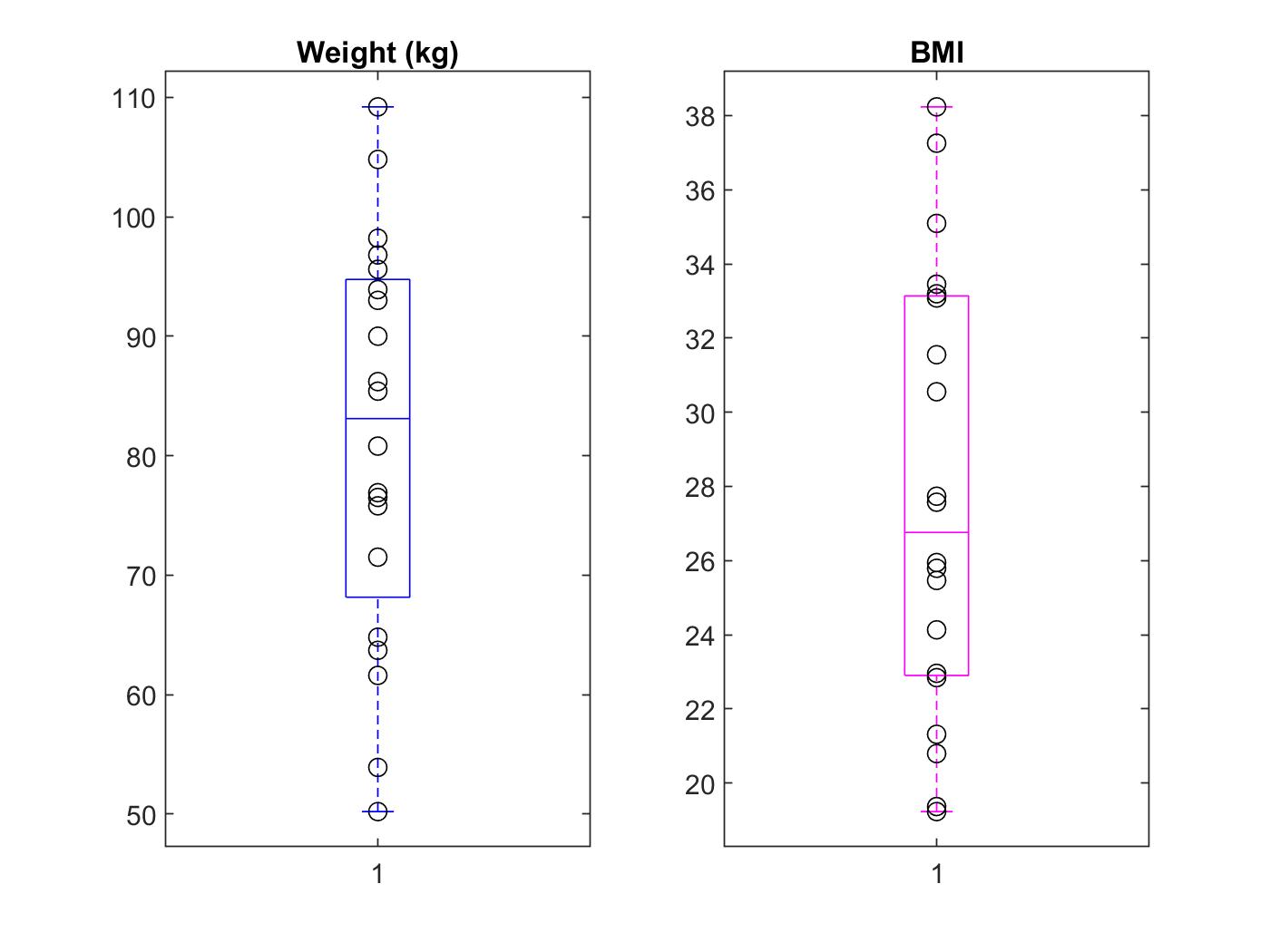  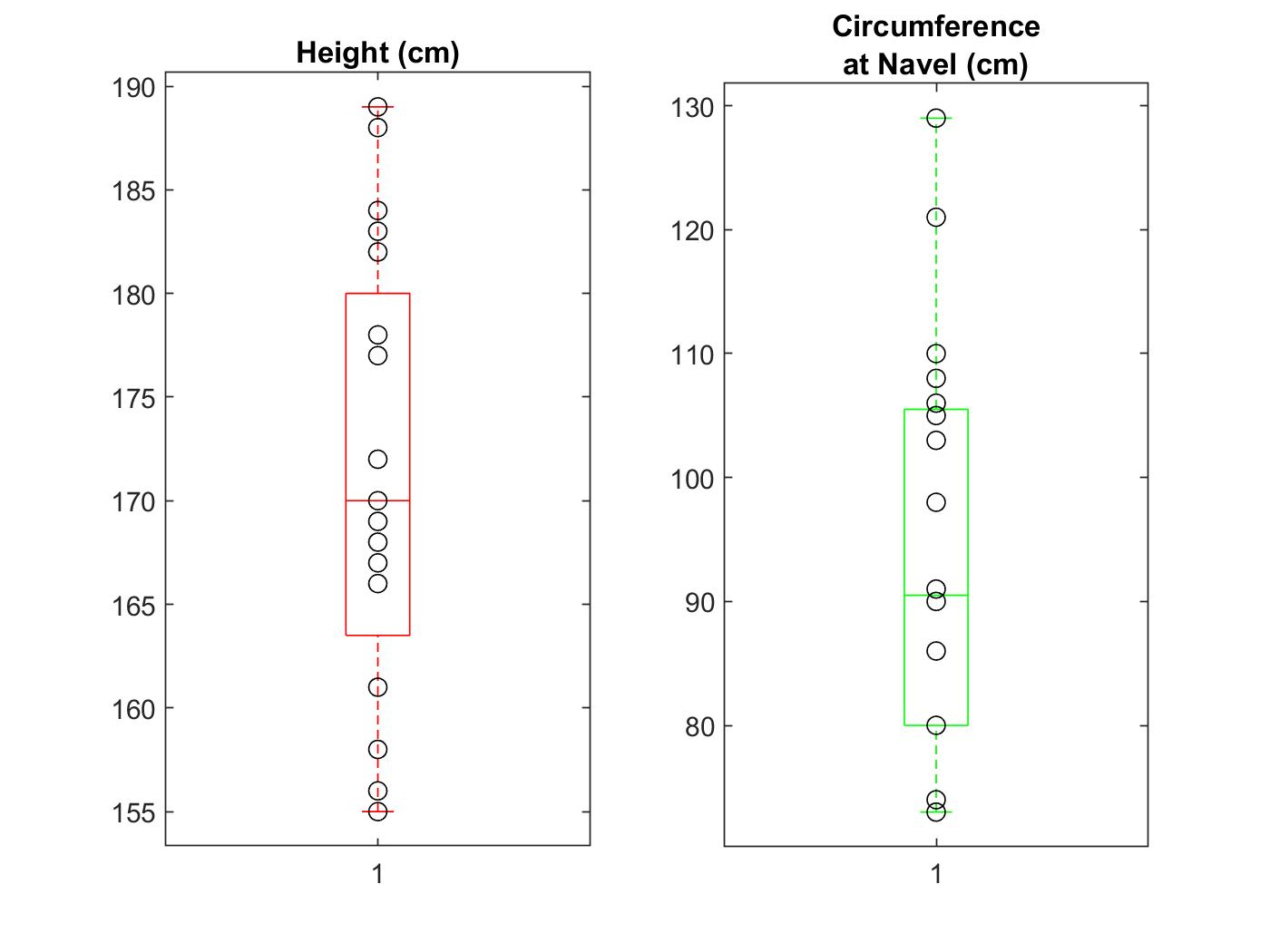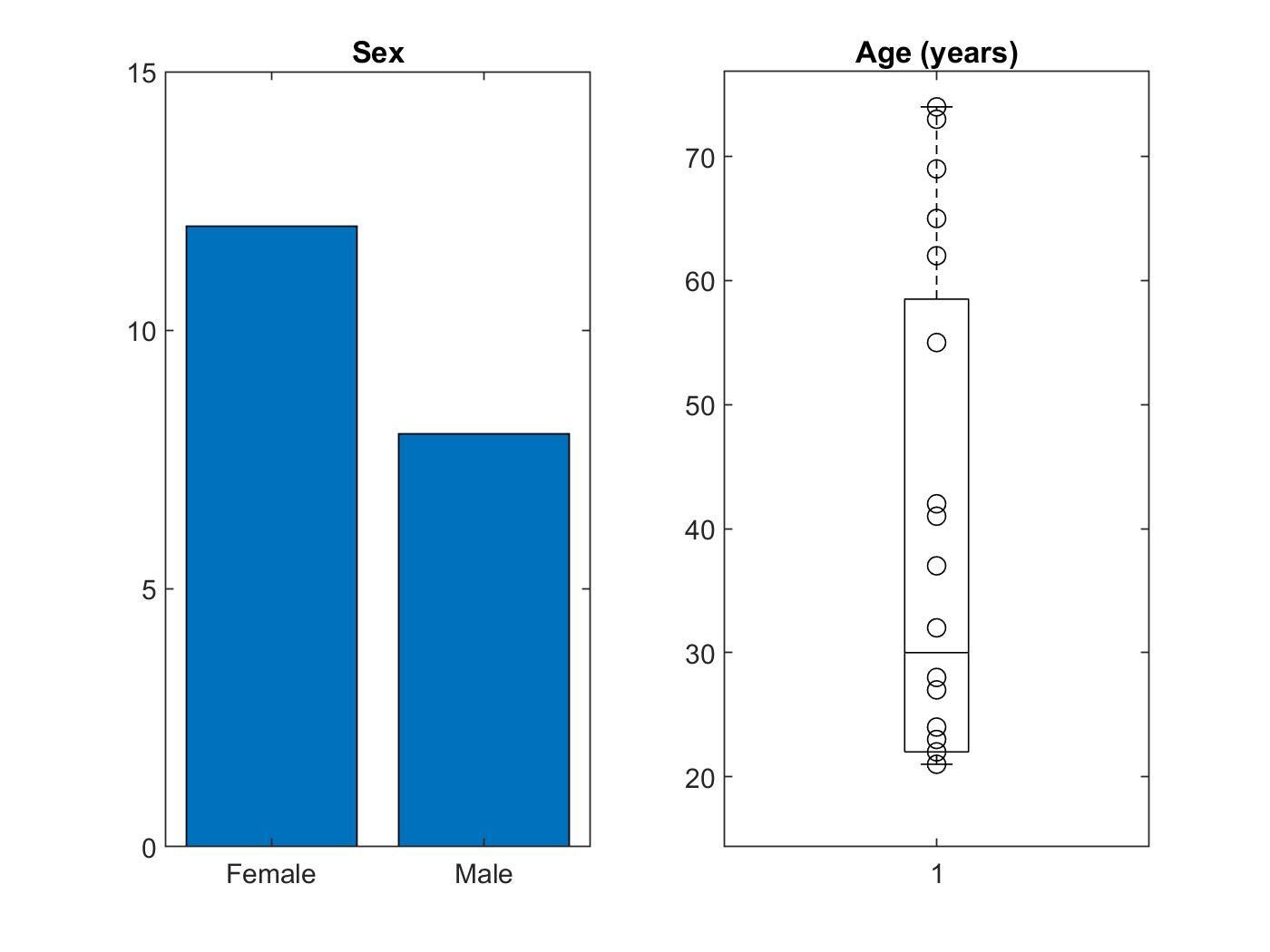 |
| --- |

Fig. S2: Physical metrics and demographics box and whisker plots with the central mark indicating the median, and the bottom and top edges of the box indicating the 25th and 75th percentiles, respectively. Plots include height (cm), weight (kg), BMI (kg/m^2^), circumference at navel (cm), sex, and age (years). The mean and standard deviation for height, weight, BMI and age are included in Table I.

| 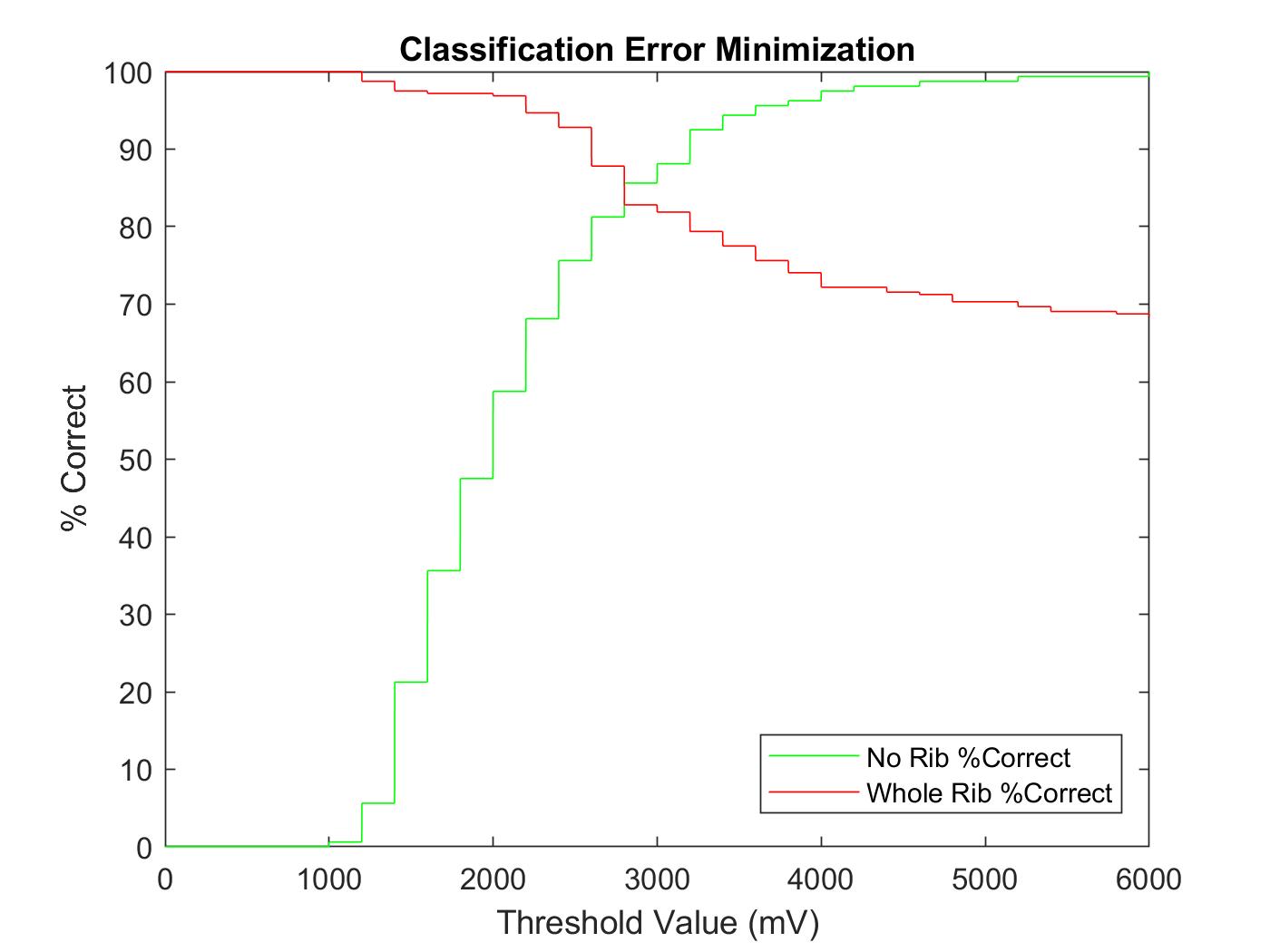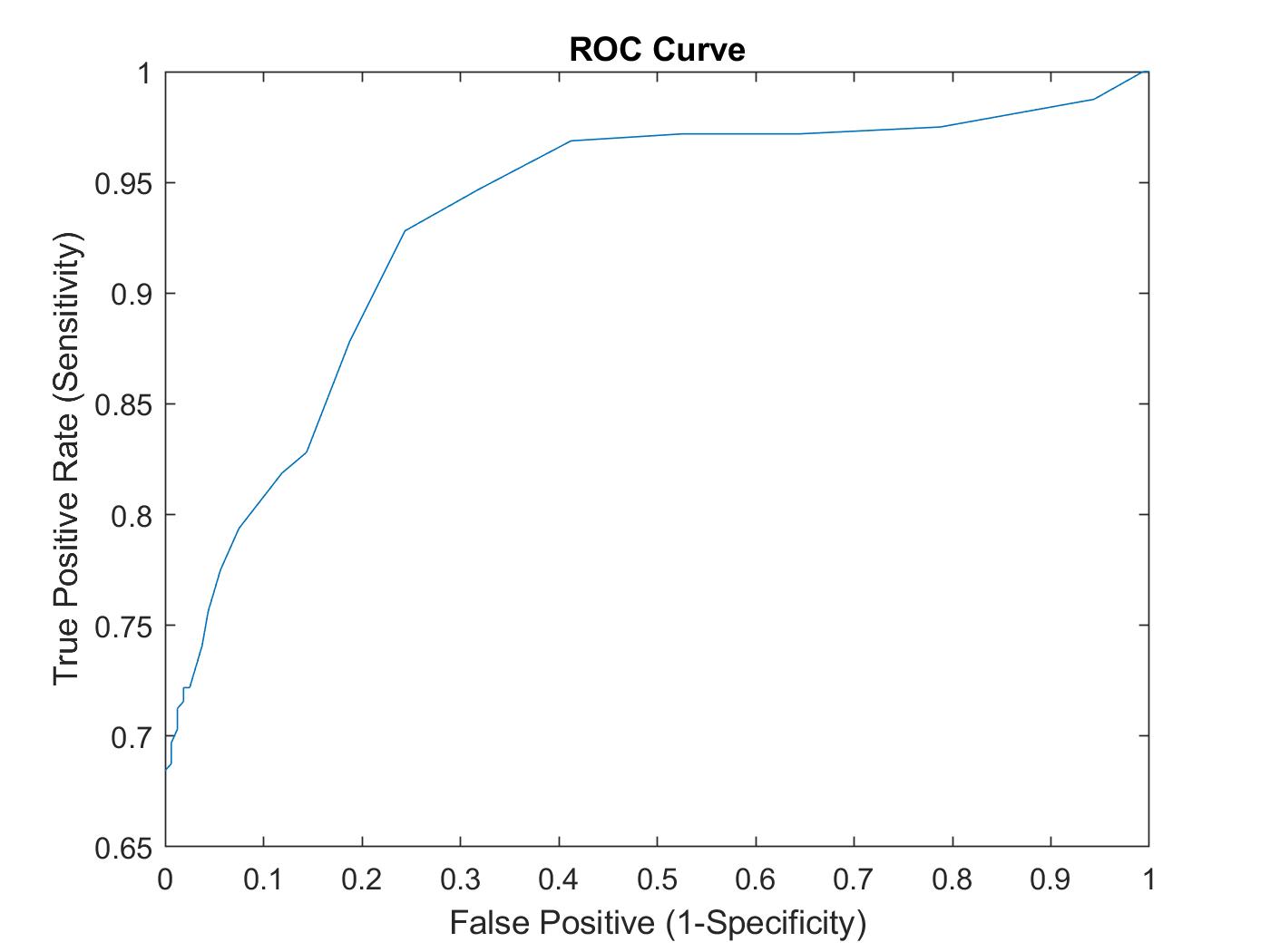 |
| --- |

Fig. S3: (Left) Example of an amplitude optimization graph for time domain thresholding for minimizing classification error. This graph corresponds to Task 1 for all participants. (Right) ROC curve (receiver operating characteristic curve) corresponding to the plot on the left.

Feature ordering and iteration analysis

The linear classification techniques performed best when all 70 features were used. To understand which features were relied on most heavily for classification, features were strategically eliminated and the model was re-trained to assess the decrease in classification accuracy. Feature order was initially assigned using the beta term (initial linear coefficient estimates) from the logistic regression model to estimate preferential weighting of the model. Top features were then rearranged and eliminated in different sequences iteratively and the models were re-trained and tested for accuracy. Finally, an ordered list of top features was obtained which corresponded to the best possible accuracy of the model for a given number of features. The same ordered list was used for both the logistic regression and SVM models to generate the Accuracy of Classification plots (Fig S4a-c). Interpretation of Fig. S4a-c is included in the main body of the manuscript.

The #1 feature was the frequency signal corresponding to bin 687.5 to 750 kHz, from the group down-sampled in bins of 10 samples by averaging over 62.5 kHz. When used alone, this feature garnered the most accurate model. This result is logical because the bin corresponds to the peak frequency of the signal. The height of this peak at around 715 kHz was the feature used to make classification decisions in the frequency-domain thresholding section. The #2 feature was participant BMI. We independently chose BMI as a key feature for separating participants into two cohorts because of its correlation with rib depth, and it is encouraging that the algorithm also ranked this feature highly. The #3 feature was the frequency signal corresponding to bin 750 to 812.5 kHz and the #5 feature was frequency bin 625 to 687.5 kHz, both from the group down-sampled in bins of 10 samples. The #4 feature was frequency bin 625 to 750 kHz, and #7 feature was frequency bin 750 to 875 kHz, both from the group down-sampled in bins of 20 samples by averaging over 125 kHz. These four features likely provide information about the height of the signal near the center frequency similar to the #1 feature. The #6 feature was participant weight. Other demographic and physical metrics information was also ranked highly: abdominal circumference at navel (#8), abdominal circumference at waist (#10), and age (#11). Overall, ten of the eleven total demographic and physical metric features appeared in the top 25 features, excluding only length from the navel to the point where chin meets neck. A few time domain features were also ranked highly. The #9 feature was the time signal corresponding to bin 32 to 36 μs, from the group down-sampled in bins of 100 samples by averaging over 4 μs. Also #13 was time bin 12-16 μs, #14 was time bin 32-40 μs (only top feature using bins of 200 samples), #17 was time bin 4-8 μs, and #18 was time bin 28-32 μs. Fig. S4d-e shows the breakdown of feature types and ranking in the top 25 features.

Frequency features were centered around the time block 562.5-875 kHz, with highest ranked features in the center of this group at 687.5-750 kHz, corresponding directly with the center frequency of the transducer and the highest point for the rib signal. Interestingly, most time features represented in the top 25 were centered around two time blocks, 4-16 μs and 24-40 μs after the electrical artifact. There was also one feature representing 56-60 μs. The distribution of the rib echo times within the rUS signal was well spread out in our dataset but 27.81% fell within the 4-16 μs range and 19.69% fell within the 24-40 μs range, which may be a reason for the high ranking of those features. Overall, the time and frequency features binned more tightly (smaller increments) were ranked higher, suggesting that higher resolution signal representation was beneficial.

The number of iterations for convergence of the logistic regression and SVM models is presented in Fig. S4f-g for the binary classification Tasks 1 and 2. Convergence data was not available for the ternary classification Task 3 which used the fitcecoc function. No constraints were defined for the number of iterations so MATLAB default values for iteration limit were used, which for the logistic regression model was 10^3^ and the SVM model was 10^6^. The logistic regression models reached convergence when the tolerance on coefficients was satisfied, and the SVM models reached convergence when the Delta Gradient tolerance was met. The logistic regression models required fewer iterations to reach convergence than the SVM models, most converging in half the iterations. Many more iterations were required for SVM model convergence in the No Rib vs. Rib classifier than the No Rib vs. Whole Rib classifier, whereas the logistic regression model used about the same number of iterations for convergence of both classifiers.

Fig. S4: Linear Classification optimal performance for reduced features by rank in terms of accuracy in (a) Task 1, (b) Task 2, and (c) Task 3. In all plots, the All Participant group is black, Low BMI is magenta, and High BMI is cyan. Logistic regression model is indicated by the asterisk marker and SVM is indicated by the triangle marker; (d) Ranking of top 25 features, separated into frequency signal features (red), time signal features (blue), and demographic features (green). (e) Further separated frequency signal features in bins of 62.5 kHz (red) and in bins of 125 kHz (magenta), as well as time signal features in bins of 4 μs (blue) and in bins of 8 μs (cyan), with same demographic features (green). (f) Number of iterations for convergence of linear model for Task 1. (g) Number of iterations for convergence of linear model for Task 2.


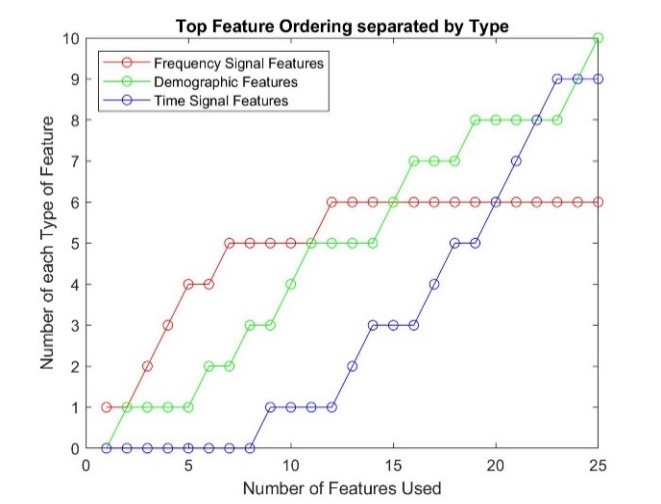


(d)


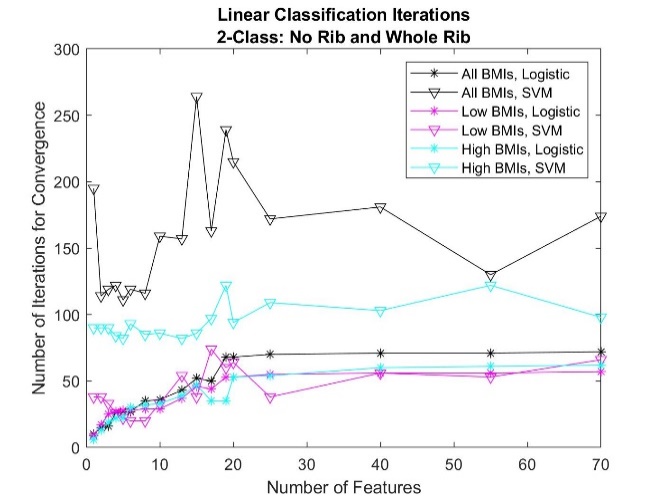


(f)


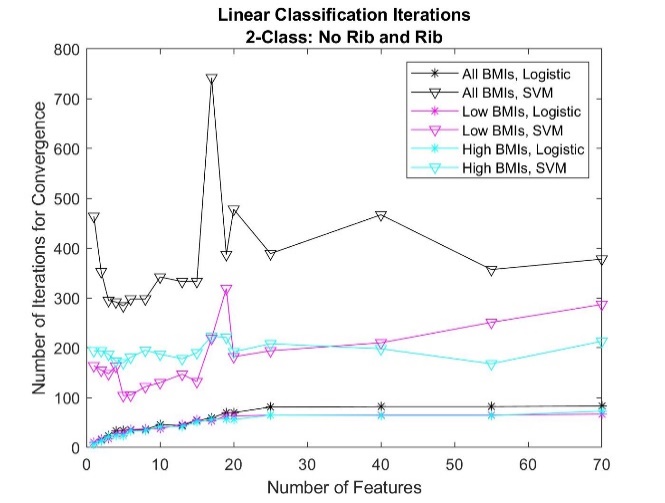


(g)


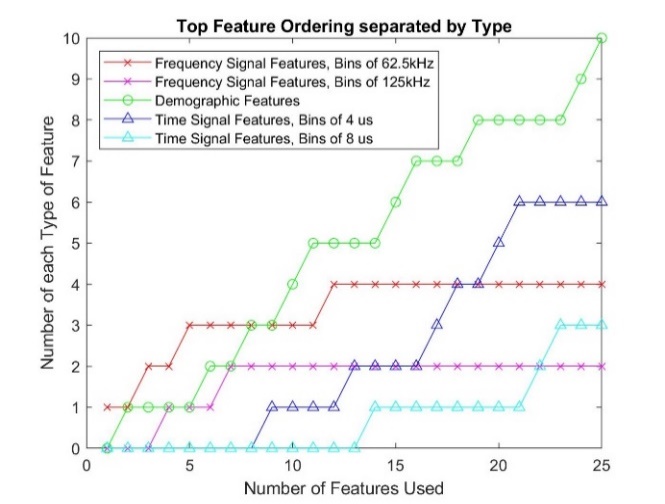


(e)


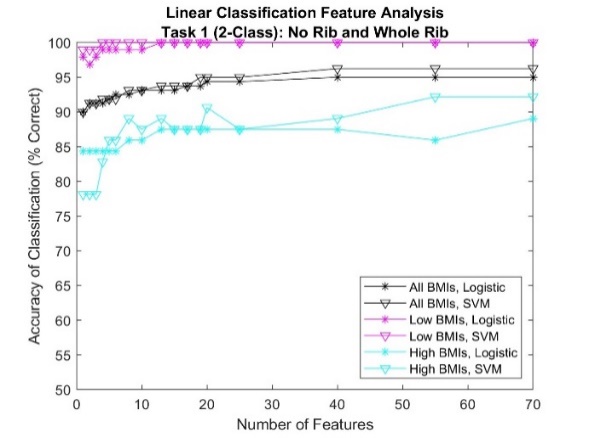


(a)


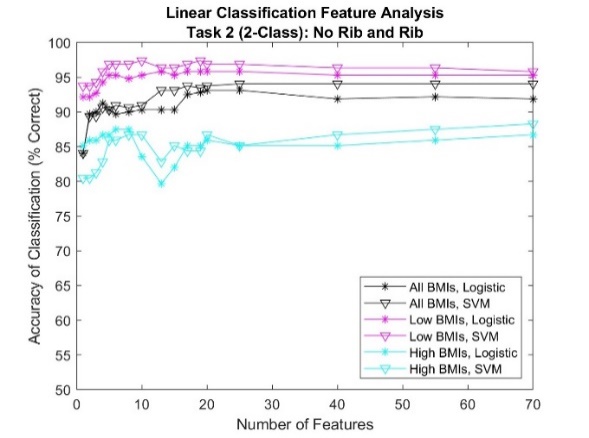


(b)


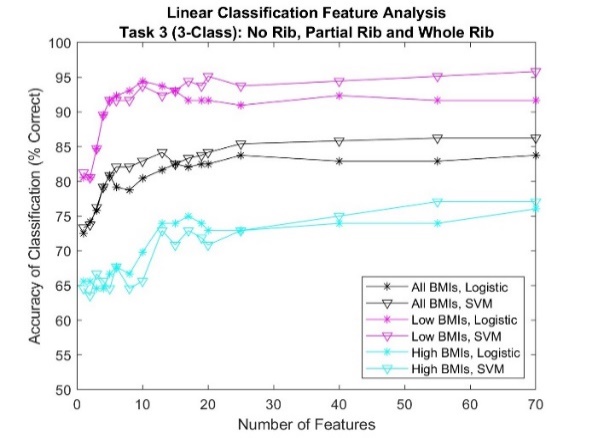


(c)

Rib Position Analysis

Analysis was performed to correlate physical metrics of body shape to rib position. The depth from the surface of the skin to the rib was measured from the cross-sectional ultrasound images acquired with a commercial imaging device (Sonosite Edge II, Fujifilm, Bothell WA, USA) and annotated using ImageJ (National Institutes of Health, Bethesda, Maryland, USA). The rib depth was also estimated independently using the rUS signals acquired in locations C and E by first calculating a “time of flight” from the time the ultrasound wave was emitted to the time the rUS signal reached the receiving transducer. This was converted into an estimated rib depth using the speed of sound in water of 1480 m/s, which is consistent with ranges cited in literature for fat, connective tissue and muscle [1],[2].

Analysis was performed to directly compare rib depth recorded from ultrasound imaging (considered ground truth) to the rib depth estimate calculated from the rUS signal time of flight, Fig. S5. The absolute value of the error between the rib depth estimate and the ground truth had a median of 0.52 cm (interquartile range 0.24-0.96). The signed value of the error was also considered to understand if overestimation or underestimation was more prevalent and if this distribution changed for participants with deeper ribs or higher BMIs. Over-estimation was indicated by positive numbers and under-estimation by negative. Higher absolute values of estimation error were recorded for deeper ground truth rib depths, and lower estimation errors were correlated with shallower ribs. Distribution of signed error (positive vs. negative) as a function of ground truth rib depth was balanced until rib depth of ~3.5 cm, and then underestimation became more prevalent. Similar trends were visible when error was plotted as a function of BMI. BMIs above 35 resulted in underestimation only, but the sign was evenly distributed until that point.

Next, the ground truth rib depth was plotted against select physical metrics features Fig. S6 (left) and similarly the rib depth estimate from the rUS signal was plotted against the same physical metric features Fig. S6 (right). Overall, the ground truth rib depth showed a cleaner linear trend and higher R-squared (indicating goodness-of-fit measure for the linear regression model) correlation with the physical metric features than the rib depth estimates. Physical metric features which had a positive correlation with ground truth rib depth included weight (R-squared=0.46), BMI (R-squared=0.80) and the ratio of abdominal circumference at the navel to height (R-squared=0.70). Rib depth estimates followed similar trends but were not as well correlated: weight (R-squared=0.15), BMI (R-squared=0.34), and abdominal circumference to height ratio (R-squared=0.37). Taken alone, height was not well correlated with rib depth (ground truth R-squared=0.13, rib depth estimate R-squared=0.10). BMI was the feature best correlated with rib depth for our 20-person participant group. It should be noted, however, that BMI may not be the best indicator of rib depth for all body shapes. Many factors lead to different distributions of tissue over the ribs which may not have been present in our small participant group. For example, people with high muscle mass often have a higher BMI but not more tissue mass in the area around the ribs. Male and female bodies carry tissue differently and may have varying amounts of tissue in this area. The position of the participant during imaging can change the distance from the device at the surface of the skin to the ribs, particularly if adipose layers are present in the area which lay differently based on gravity. The abdominal circumference to height ratio was an attempt to provide a more direct metric to account for these body shapes, but did not perform as well as BMI.

| 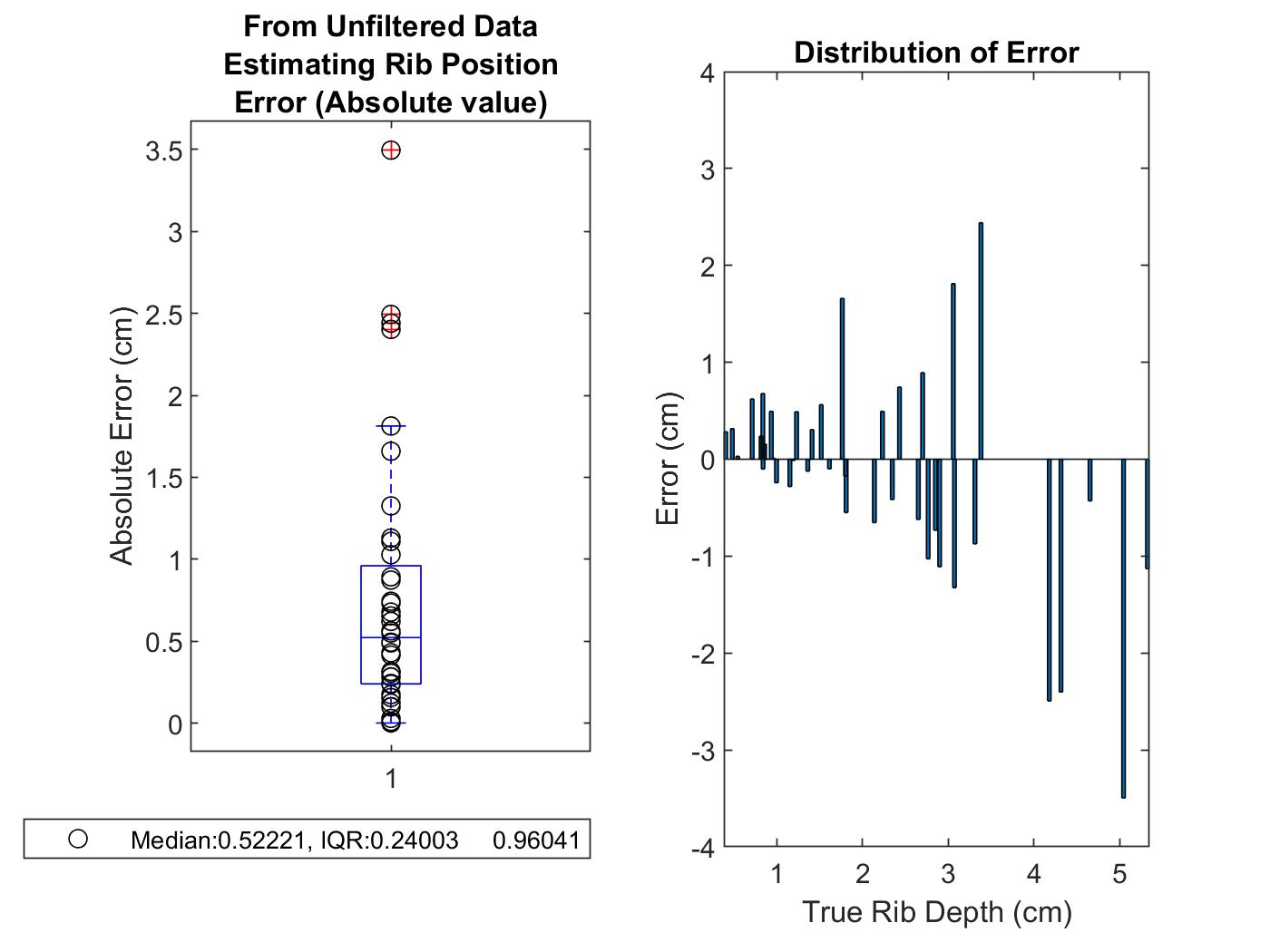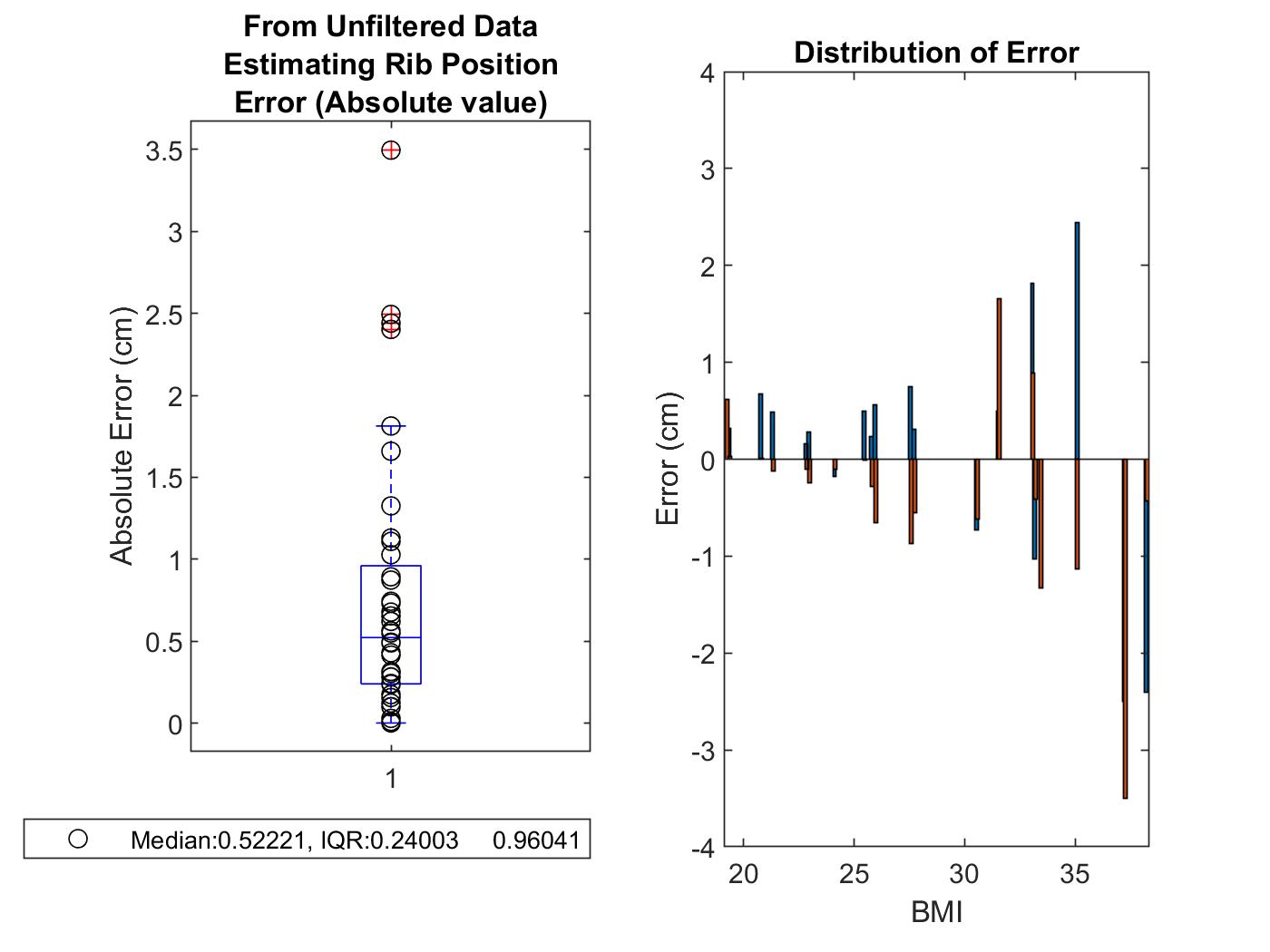  (b)  (c)  (a) |
| --- |

Fig. S5: (a) Box and whisker plot of the absolute value of the error between the rib depth estimate and the ground truth. (b) Distribution of error as a function of the ground truth rib depth. (c) Distribution of error as a function of the BMI.

| Physical metric features vs. ground truth rib depths measured from cross-sectional images | Physical metric features vs. Rib depth estimate |
| --- | --- |
| 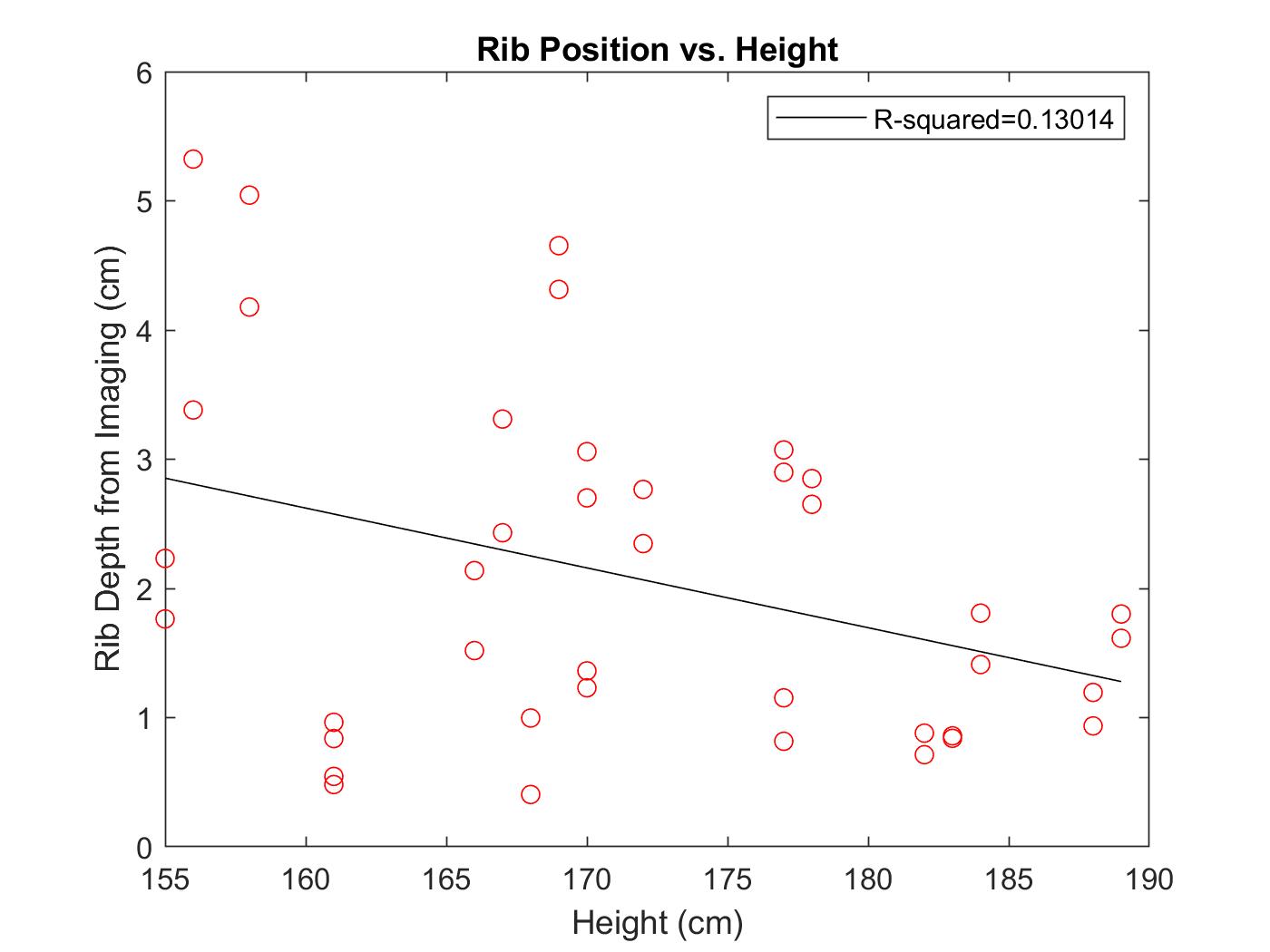 | 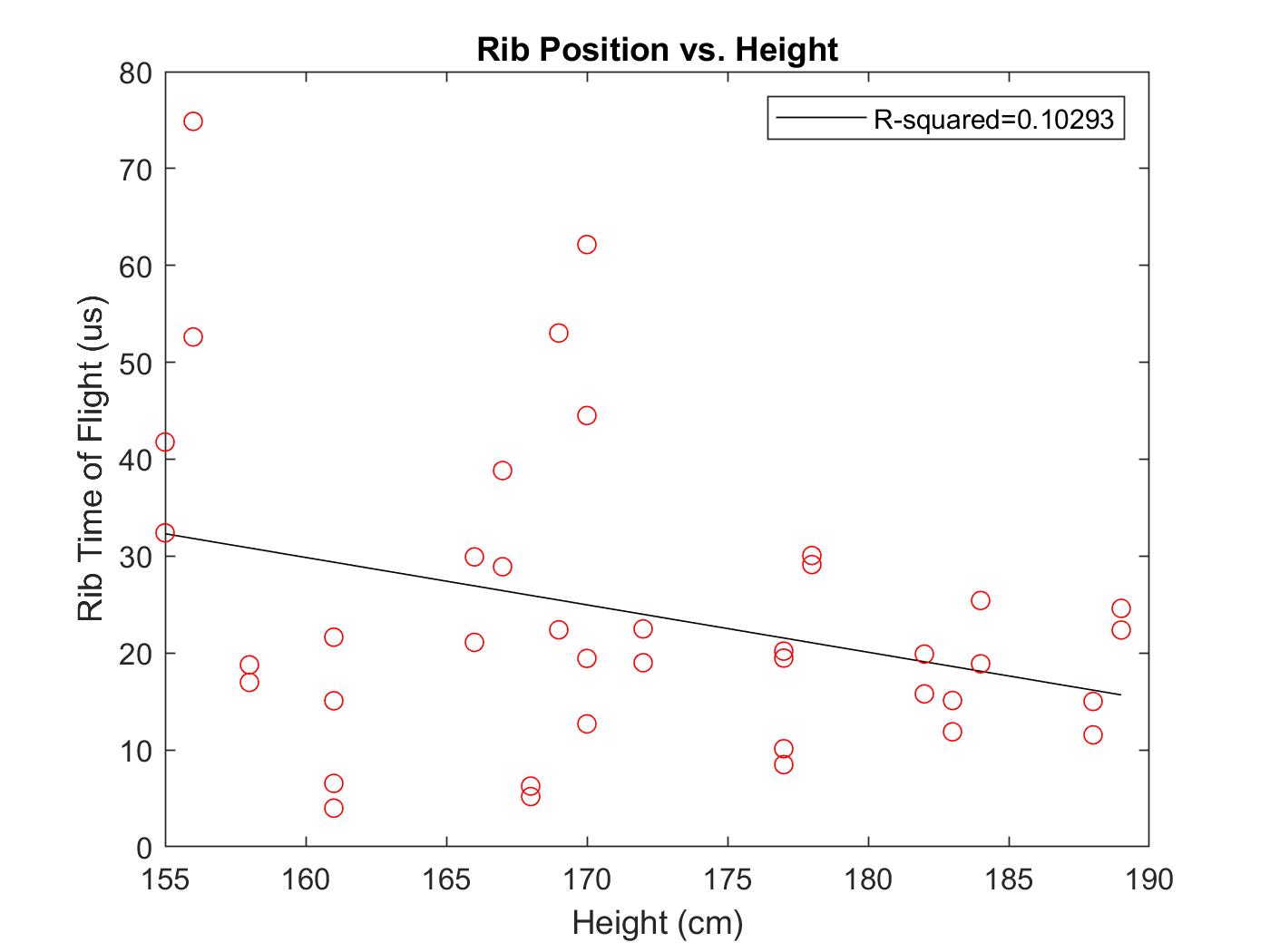 |
| 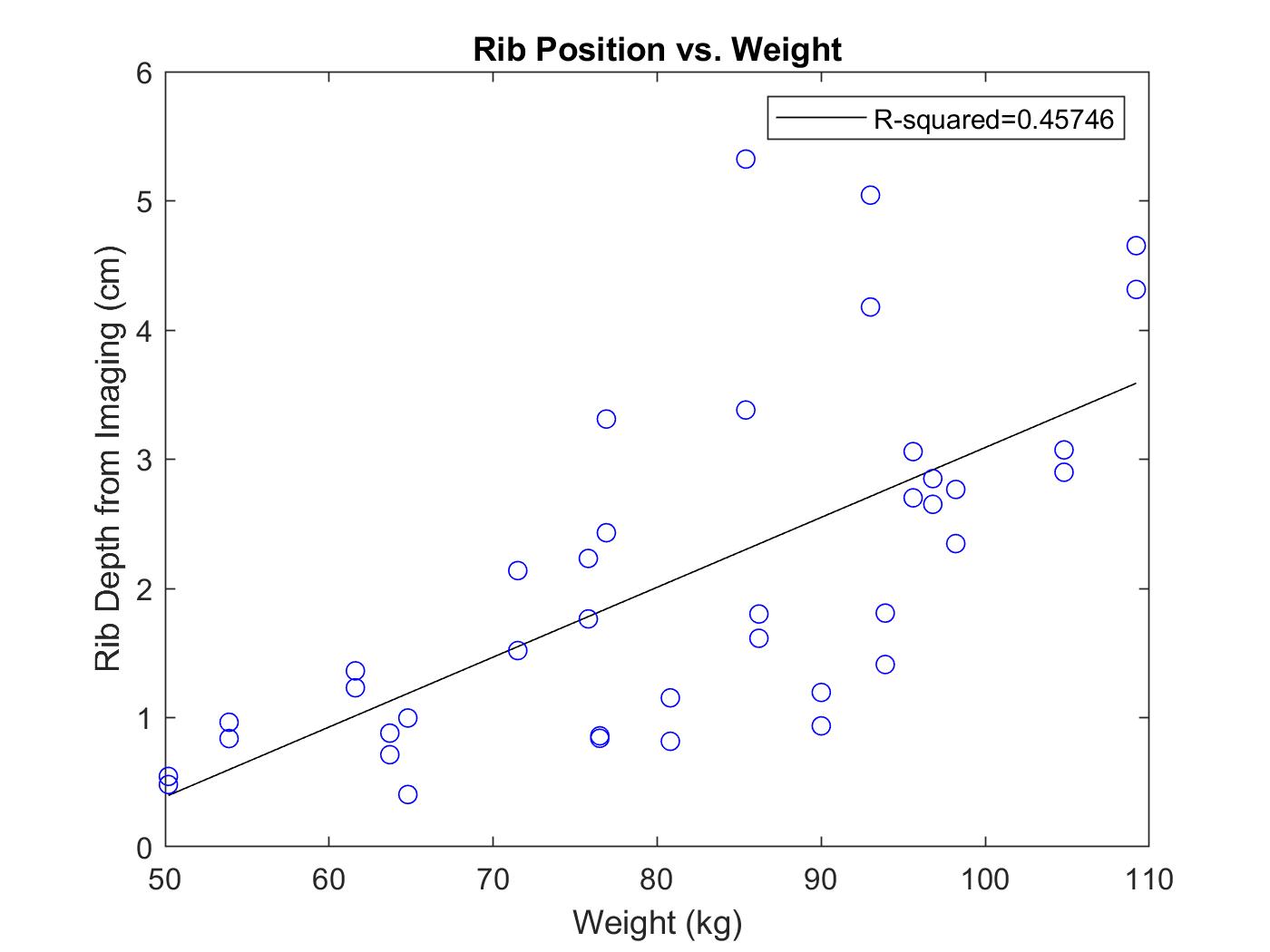 | 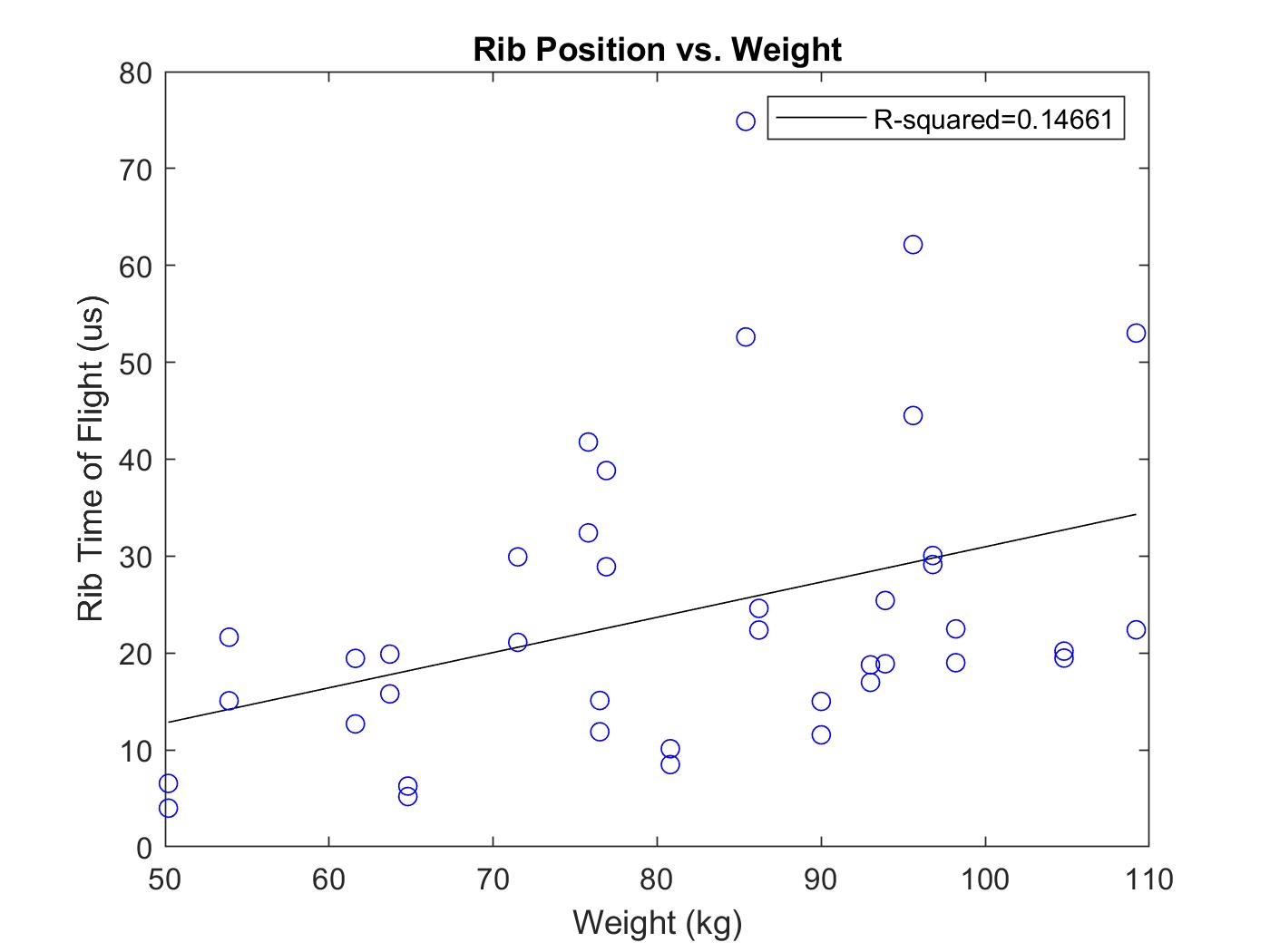 |
| 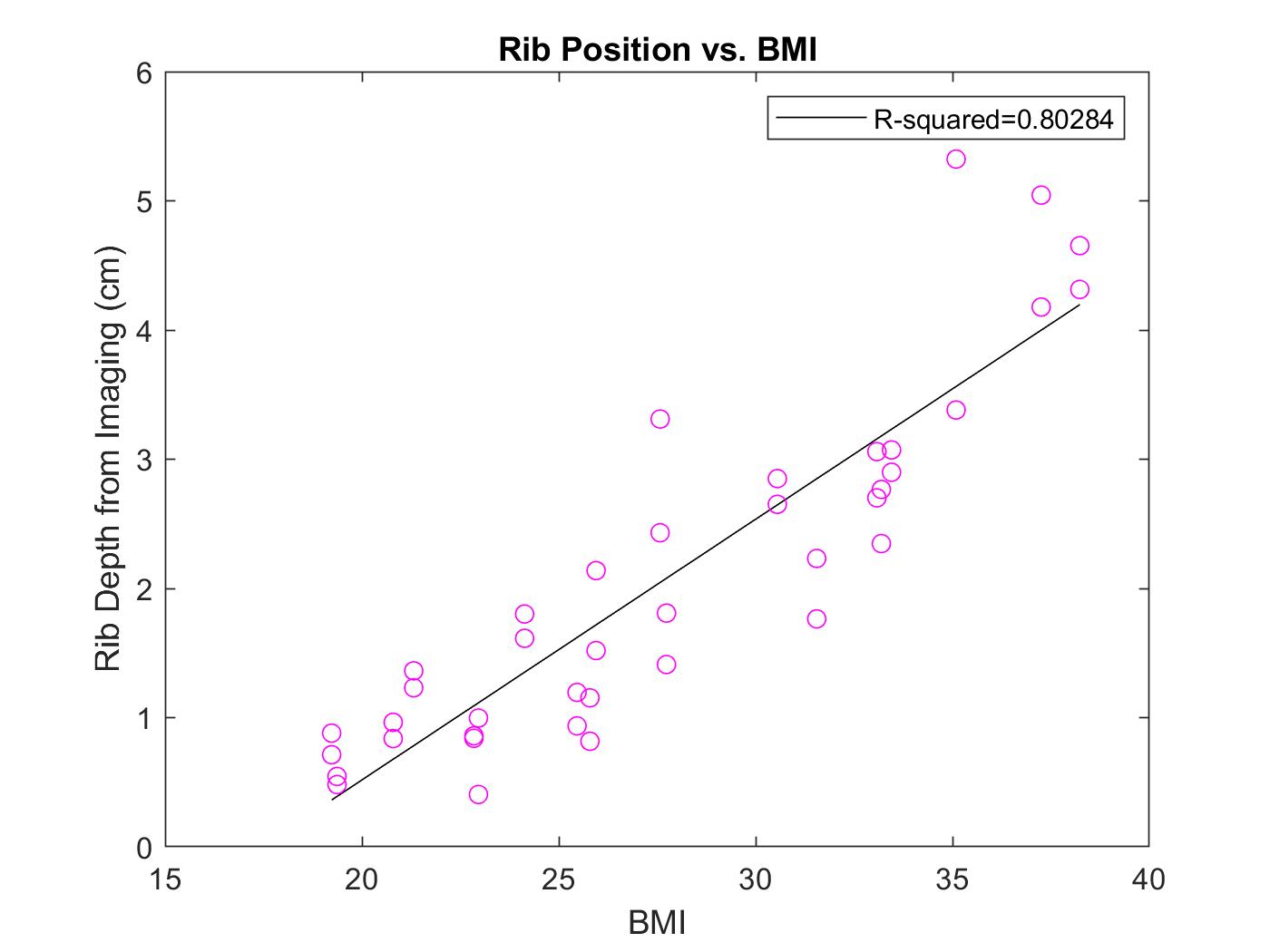 | 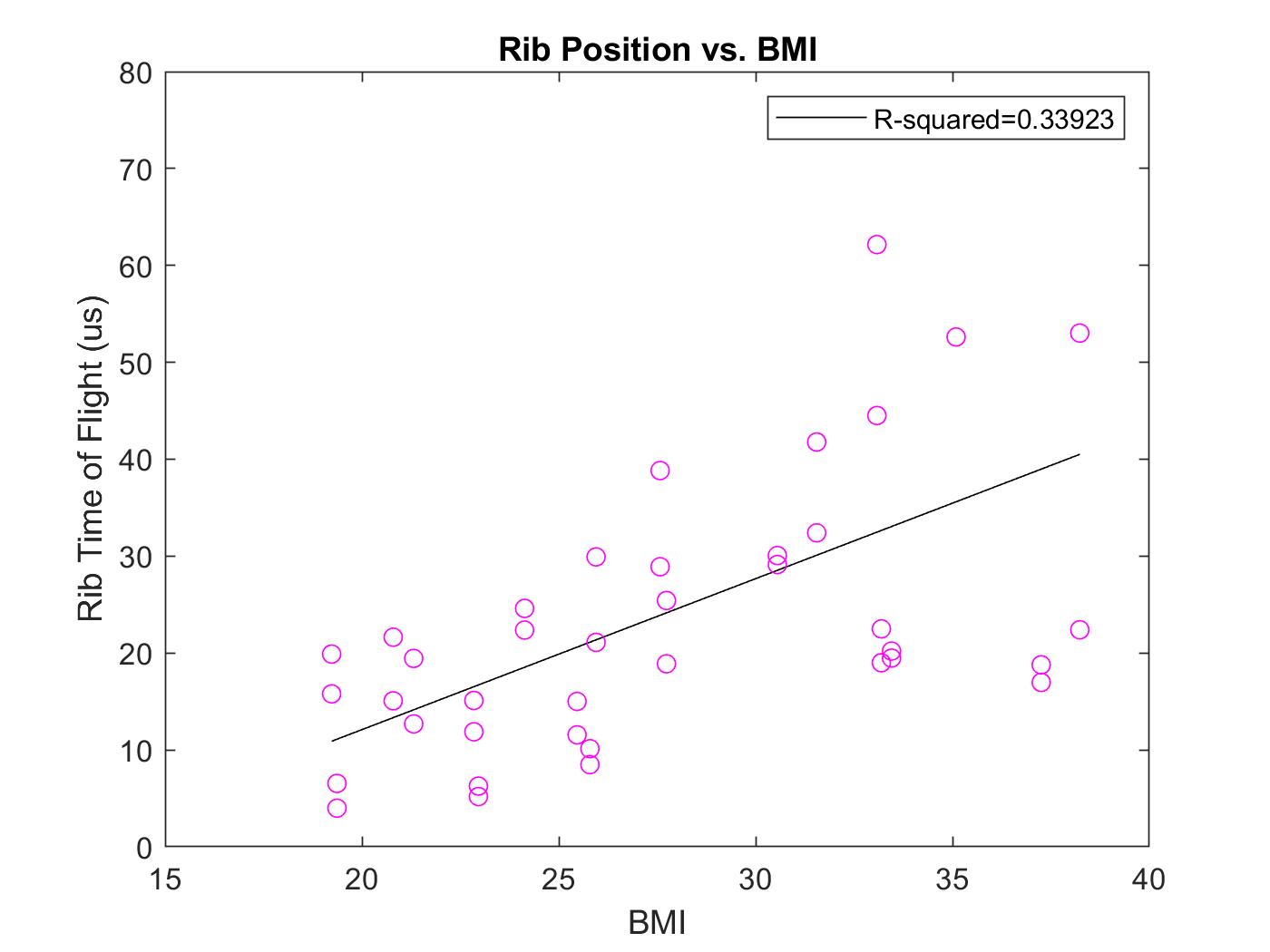 |
| 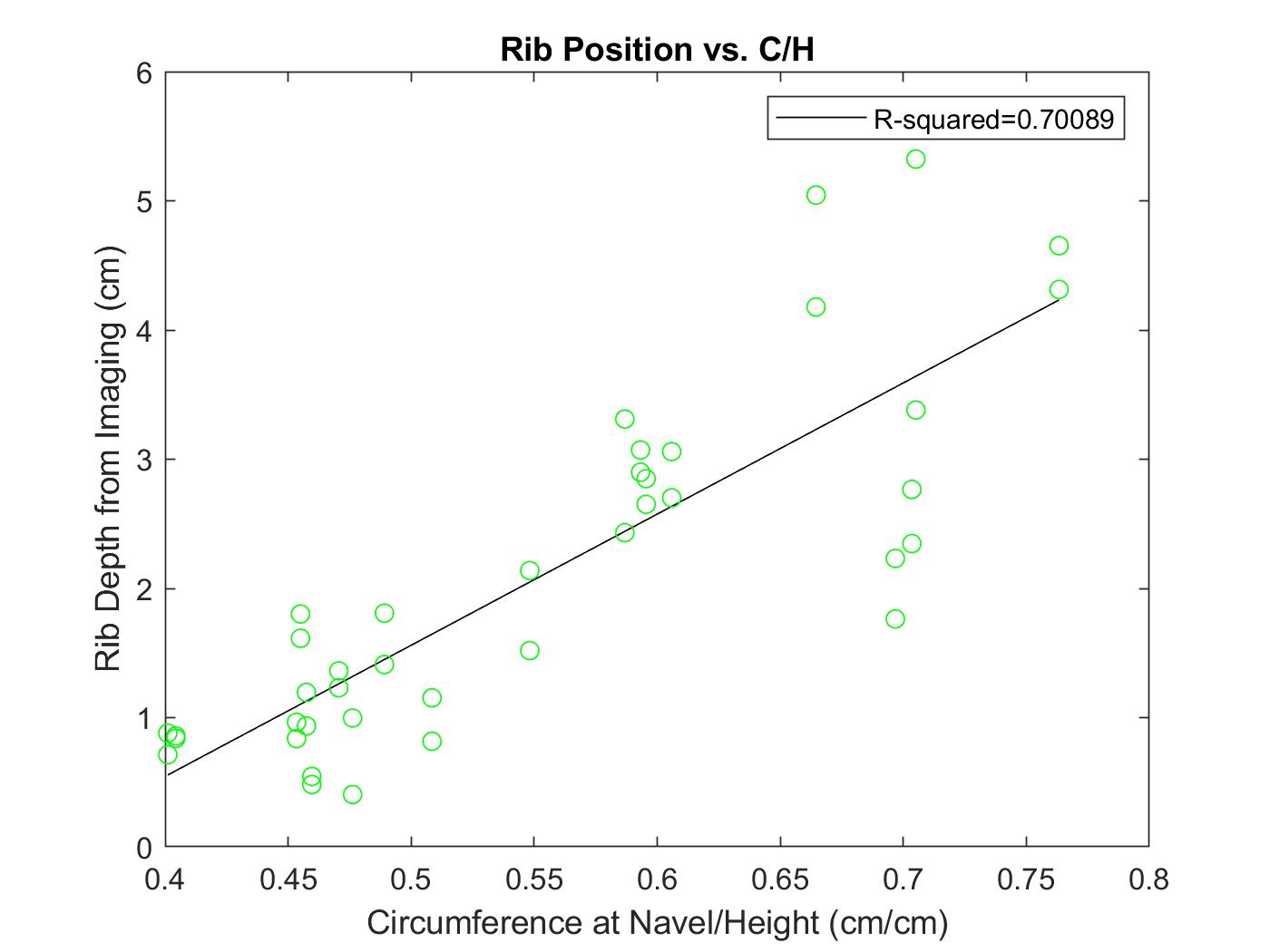 | 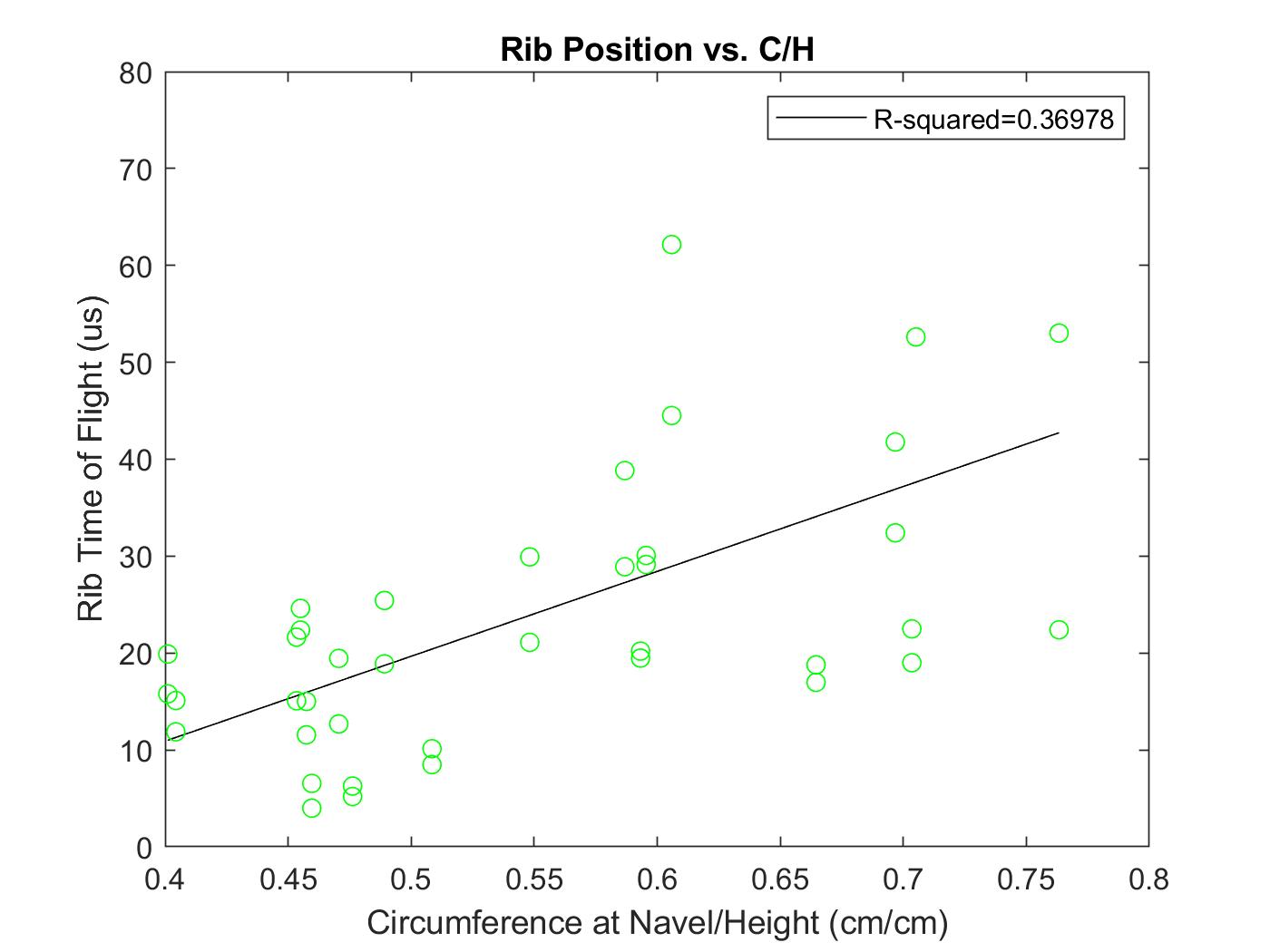 |

Fig. S6: (Left column) Ground truth rib depth measured from ultrasound images plotted against physical metric features. (Right) Rib depth estimate from the rUS signal plotted against physical metric features. Physical metric features are (top to bottom): Height (cm), Weight (kg), Body Mass Index (BMI) (kg/m^2^), and ratio of abdominal circumference at the navel to height (C/H) (cm/cm).

Notes on Signal Processing Decisions

A number of approaches were attempted to increase the performance of the time domain amplitude thresholding method described in the Methods section. Though not ultimately implemented, documentation of these approaches may prove useful for avoiding pitfalls in future applications. For Task 1, a template-based subtraction method was initially performed instead of the windowing approach to eliminate the electrical artifact. Subtracting a template of the electrical artifact signal and then applying a lowpass filter (1300kHz) to eliminate high-frequency noise left by the artifact was quite successful in reducing the amplitude of the large electrical artifact in the first 15μs of the signal but beyond that time point, the subtraction tended to cause distortion of the original signal which could lead to erroneous classification as a rib signal. Therefore, it was decided that windowing to eliminate the electrical artifact was preferable because it was less computationally expensive and less prone to introducing signal distortion. For Task 3, another approach for setting the second threshold separating the Partial rib class from the Whole rib class was to use a time-dependent separator instead of a second amplitude threshold. In this approach, the width of the signal that was above Threshold 1 was calculated and a threshold width was identified that best separated the Partial rib class from the Whole rib class. This algorithm can also be implemented in real time with analog electronics so it would be fit for the simplified application presented in this first model but was found to be less accurate than the amplitude-dependent separator and was ultimately abandoned.

Similarly, another approach attempted in the frequency domain amplitude thresholding was not windowing in the frequency domain after the FFT was performed. The results in the All Participants and Low BMI group were similar, however classification accuracy decreased in the High BMI subgroup by as much as 6%. This is because the frequency domain rUS signals for High BMI participants sometimes had lower frequency signal peaks at ~400 kHz which appeared in both the Rib and No Rib conditions that surpassed the height of the genuine rib signal which appeared at ~715 kHz. This ~400 kHz signal may have been caused by ultrasound reflection off additional tissue layers and resulted in false positive Rib classifications if not windowed out.

Reference List

[1] C. CRS. Foundations of Biomedical Ultrasound. Oxford University Press; 2007.

[2] Azhari H. Appendix A. In: Basics of Biomedical Ultrasound for Engineers. Hoboken, NJ: John Wiley &amp; Sons; 2010.
